# Protein intake and outcome of critically ill patients: analysis of a large international database using piece-wise exponential additive mixed models

**DOI:** 10.1186/s13054-021-03870-5

**Published:** 2022-01-11

**Authors:** Wolfgang H. Hartl, Philipp Kopper, Andreas Bender, Fabian Scheipl, Andrew G. Day, Gunnar Elke, Helmut Küchenhoff

**Affiliations:** 1 Department of General, Visceral, and Transplantation Surgery, University Medical Center, Campus Grosshadern, LMU Munich, Marchioninistr. 15, 81377 Munich, Germany; 2 Statistical Consulting Unit, StaBLab, Department of Statistics, LMU Munich, Munich, Germany; 3 Department of Statistics, LMU Munich, Munich, Germany; 4 Workgroup of Functional Data Analysis, Department of Statistics, LMU Munich, Munich, Germany; 5 Clinical Evaluation Research Unit, Kingston Health Sciences Centre, Kingston, ON Canada; 6 Department of Anaesthesiology and Intensive Care Medicine, University Medical Center Schleswig-Holstein, Campus Kiel, Kiel, Germany

**Keywords:** Critical care, Nutrition, Protein supply, Survival

## Abstract

**Background:**

Proteins are an essential part of medical nutrition therapy in critically ill patients. Guidelines almost universally recommend a high protein intake without robust evidence supporting its use.

**Methods:**

Using a large international database, we modelled associations between the hazard rate of in-hospital death and live hospital discharge (competing risks) and three categories of protein intake (low: < 0.8 g/kg per day, standard: 0.8–1.2 g/kg per day, high: > 1.2 g/kg per day) during the first 11 days after ICU admission (acute phase). Time-varying cause-specific hazard ratios (HR) were calculated from piece-wise exponential additive mixed models. We used the estimated model to compare five different hypothetical protein diets (an exclusively low protein diet, a standard protein diet administered early (day 1 to 4) or late (day 5 to 11) after ICU admission, and an early or late high protein diet).

**Results:**

Of 21,100 critically ill patients in the database, 16,489 fulfilled inclusion criteria for the analysis. By day 60, 11,360 (68.9%) patients had been discharged from hospital, 4,192 patients (25.4%) had died in hospital, and 937 patients (5.7%) were still hospitalized. Median daily low protein intake was 0.49 g/kg [IQR 0.27–0.66], standard intake 0.99 g/kg [IQR 0.89– 1.09], and high intake 1.41 g/kg [IQR 1.29–1.60]. In comparison with an exclusively low protein diet, a late standard protein diet was associated with a lower hazard of in-hospital death: minimum 0.75 (95% CI 0.64, 0.87), and a higher hazard of live hospital discharge: maximum HR 1.98 (95% CI 1.72, 2.28). Results on hospital discharge, however, were qualitatively changed by a sensitivity analysis. There was no evidence that an early standard or a high protein intake during the acute phase was associated with a further improvement of outcome.

**Conclusions:**

Provision of a standard protein intake during the late acute phase may improve outcome compared to an exclusively low protein diet. In unselected critically ill patients, clinical outcome may not be improved by a high protein intake during the acute phase.

*Study registration* ID number ISRCTN17829198

**Supplementary Information:**

The online version contains supplementary material available at 10.1186/s13054-021-03870-5.

## Introduction

For critically ill patients, current guidelines recommend a high protein intake early during critical illness. According to the American Society of Parenteral and Enteral Nutrition (A.S.P.E.N.), more than > 80% of protein targets of 1.2–2.0 g/kg day should be administered within 48–72 hours, and with an even higher target in burns and trauma patients [1]. Guidelines of the European Society of Parenteral and Enteral Nutrition (ESPEN) advise to deliver progressively up to 1.3 g/kg day protein during the acute phase [2]. Quality of evidence for both recommendations, however, is low; recently, a panel of experts was unable to make a clear recommendation for protein intake in the early (acute) phase of critical illness [3].

The literature addressing the relationship between protein intake and outcome of critically ill patients reveals divergent results. The vast majority of observational studies found a significant association between an early high protein intake and better outcome [4], whereas some post hoc analyses of randomized studies suggested the opposite [5, 6]. Several meta-analyses evaluated protein intake in randomized studies originally examining the effect of a different calorie intake; these analyses showed that protein intake was largely unimportant for outcome [7–10].

Thus far, there is no adequately powered randomized multicenter trial on the effect of protein intake on outcome in critically ill patients, and observational studies have been criticized because of numerous methodological weaknesses [11, 12]. This analysis of a large international database was designed with the aim of better understanding the associations between protein intake and the rate of in-hospital death and live hospital discharge.

## Methods

### Study overview

#### Database

The present study is an analysis of a subset of a large international point prevalence survey of nutrition practice in intensive care units (ICUs) (www.criticalcarenutrition.com/ins) conducted in 2007, 2008, 2009, 2011, 2013 and 2014. Details of the survey are provided in the Additional File and elsewhere [13]. In total, 21,100 adult patients from 785 ICUs had been included into the survey.

#### Data collection

Using a secure web-based data collection tool, the following information was collected: date of ICU admission, admission category (elective surgery, emergency surgery, medical), primary admission diagnosis (nine categories), sex, age, body mass index (BMI), duration of mechanical ventilation/propofol therapy, and APACHE II score on admission day. Treating physicians recorded daily the amount of calories, and type (enteral, parenteral) and amount of amino acids or protein received from parenteral nutrition (PN) and/or enteral nutrition (EN). Daily protein intake was collected from the day of ICU admission (partial day) to a maximum of additional 11 days after admission date. In the current analysis, we ignored protein intake received on the day of ICU admission, and referred to the subsequent discrete calendar days as “day on diet *#1 to #11”*.

For the first three days on diet, we recorded the number of days on which a patient had, at any time, been mechanically ventilated, or had received PN or oral feeding. Patients were followed for a maximum of 60 days after ICU admission. We registered time until in-hospital death or live hospital discharge. Patients remaining alive in hospital for more than 60 days were considered censored for either risk at that time.

A patient’s continuous survival time or time until live hospital discharge was calculated as “*days after ICU admission”*, where a “day” was defined as a 24-h period starting on the exact date and time of ICU admission. Consequently, days on diet, and days after ICU admission differed to the extent that the former always started/ended at 12 a.m. (midnight), whereas the latter could start/end at any time of the previous calendar day.

### Patients

#### Inclusion and exclusion criteria

Patients extracted from the database were ≥ 18 years of age and had a BMI > 13 kg/m^2^. They had been treated in the ICU for at least 96 h. In addition, on at least one day during the first 96 h of their ICU stay, included patients received medical nutrition therapy (MNT) (enteral or parenteral), and mechanical ventilation.

#### Quantifying protein intake

Total daily protein intake was classified by using established thresholds [14] defining three different levels based on the amount of received protein (level I, low: < 0.8 g/kg per day; level II, standard: 0.8–1.2 g/kg per day; level III, high: > 1.2 g/kg per day). For categorization, we first calculated daily protein intake by summing up protein intake from EN, and amino acid intake from PN. For amino acids, we used a correction factor of 0.83 to equivalently convert amino acid intake to that of protein [15]. Since in critically ill patients oral intake of protein is rare and fairly low [16], we calculated daily protein intake only from PN or EN irrespectively of whether or not there had been additional oral intake. Days with an exclusively oral intake were ranked among days with the lowest protein intake level (< 0.8 g/kg per day).

Registration of protein intake was stopped, if a patient had died or had been discharged from the ICU, or had been in the ICU for more than 11 days. Our statistical model, however, required for all surviving patients information on protein intake on days on diet #1 to #11 after ICU admission, even if patients had been discharged from the ICU before day #11 [17]. For those patients we imputed a daily standard protein intake (level II, range 0.8–1.2 g/kg per day) between the day of ICU discharge and day on diet #11 [18].

### Statistical analysis

We used piece-wise exponential additive mixed models allowing an easy accommodation of time-varying covariates such as nutrition [19]. These models had been recently extended to cumulative effects [20, 21] and to competing risks [22] to estimate cause-specific hazard rates beyond day three after ICU admission for a) time until in-hospital death and b) time until live hospital discharge (competing risk analysis). Full details are provided in the Additional File.

Our model considered several confounder variables, including the route of nutrient supply (number of days with oral or parenteral nutrition), number of days with mechanical ventilation or with propofol therapy, year of therapy, Apache II score at admission, admission diagnosis, admission category (surgical elective/emergency, medical), age, gender, BMI and a random ICU effect.

When analyzing associations of protein intake with the rate of in-hospital death and live hospital discharge, we used a time lag of 4 days (*lag-time*) to minimize the indication bias originating from possible changes of protein intake just prior to the event. Furthermore, specific metabolic/nutritional interventions may require a corresponding time span to become effective [23, 24].

In addition, we used a *lead time* which was defined as twice the number of days a patient had had a medical nutrition therapy, and which should account for the fact that a short duration of medical nutrition therapy is unlikely to affect outcome throughout prolonged periods of time. These lag and lead times constituted a time window in which protein intake on a specific day on diet could have affected subsequent hazards of in-hospital death/live hospital discharge. For the primary analysis, definition of the time window was such that the length of the lead period depended on the number of days a patient had received MNT (*dynamic time window*, see Additional file 1: Figure S1).

To facilitate the interpretation of the associations between protein intake and outcome as estimated by our model, we constructed five different hypothetical protein diets (reflecting three different levels of daily protein intake (low, standard, high) during days on diet #1 to #11 (Table 1). These intake levels were also used to model associations between nutrition and outcomes. Similar to Koekkoek et al. [25], we differentiated between an early (days on diet #1 to #4) and a late (days on diet #5 to #11) acute phase.

**Table 1:** Definition of hypothetical protein diets. Number of days with a defined level of medical nutrition therapy starts with day 1 after ICU admission. On days not specified, protein intake was identical with that of the comparison diet

Diet	Definition
Exclusively low protein diet	< 0.8 g protein/kg per day (level I, median 0.49 g/kg day) on days on diet #1 to #11
Late standard protein diet	0.8–1.2 g protein/kg per day (level II, median 0.99 g/kg day) on days on diet #5 to #11
Early standard protein diet	0.8–1.2 g protein/kg per day on days on diet #1 to #11
Late high protein diet	> 1.2 g protein/kg per day (level III, median 1.41 g/kg day) on days on diet #5 to #11
Early high protein diet	> 1.2 g protein/kg per day on days on diet #1 to #11

Protein diets represented artificial concepts similar to clinically established nutrition protocols, and they did not reflect selected patient cohorts contained in this study. All hazard ratios (pairwise comparisons of different hypothetical protein diets) were calculated under the ceteris paribus assumption.

Subsequently, we used our model to compare expected outcomes from these hypothetical protein diets while controlling for confounders. We designed six different pairwise comparisons of these artificial diets analyzing in-hospital mortality and live hospital discharge. We estimated the cause-specific, time-varying hazard rates associated with these diets in comparison with each other. Furthermore, we calculated absolute risks (cumulative incidence functions) and subdistribution proportional hazards models [26].

We also performed two sensitivity analyses. To consider a potential bias by attributing a daily standard protein intake to those patients who had been discharged alive from the ICU before day 11 after ICU admission, we repeated our analysis using in-ICU death and live ICU discharge as competing risks. Analysis of ICU outcomes did not require assumptions regarding protein intake after ICU discharge. For the second sensitivity analysis, we used a time window which was not dynamic but static (lag-lead time: 4 and 60 days, respectively) assuming alternatively that associations between protein intake and outcome did not depend on the duration of MNT.

Finally, we performed two subgroup analyses: for the first, we selectively analyzed outcomes in those patients having a BMI > 30 kg/m^2^ at ICU admission. The second subgroup analysis was intended to identify a potential interrelationship between the total calorie intake and associations between protein intake and outcomes. For that subgroup analysis, we first expressed total energy intake on day three after ICU admission as a fraction of the daily caloric target calculated at ICU admission. Ways to calculate this target were left to the judgment of the individual provider. Then, a specific nutritional category was assigned to this fraction defining three different subgroups (low calorie intake: < 30% of target, moderate: 30–70% of target, high: > 70% of target).

The statistical programming environment R was used for visualization and data analysis. The models were estimated using the R packages pammtools [27] and mgcv [28].

## Results

### Study participants

**Fig. 1:**
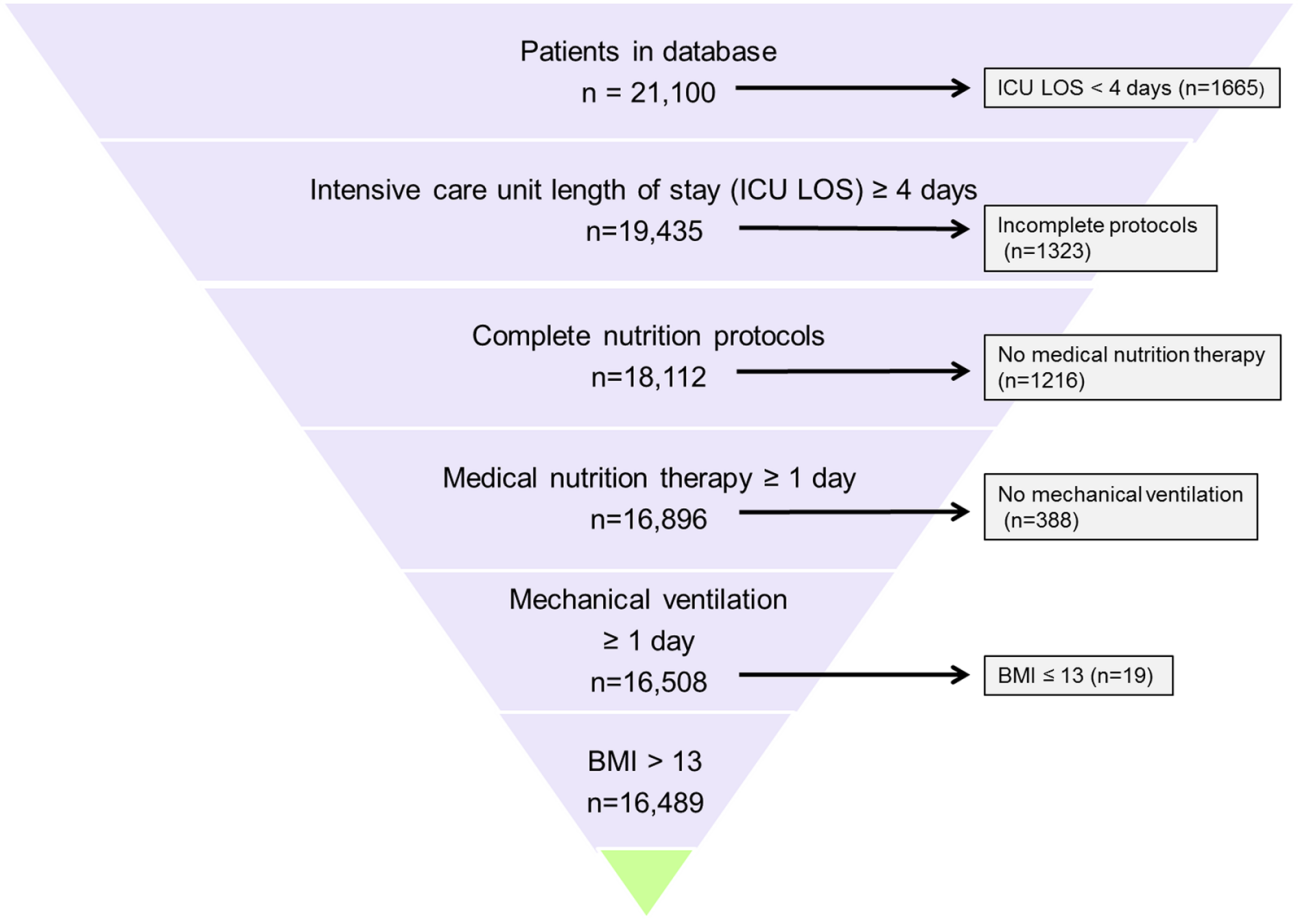
Participant flow chart

Protein intake was assessed in 16,489 patients who met our inclusion criteria (Fig. 1). Of these patients, 3366 patients (20.4%) died in hospital after 96 h but within 28 days after ICU admission, 4186 patients (25.4%) died in hospital after 96 h but within 60 days after ICU admission. 11,346 patients were discharged alive within 60 days after ICU admission. After 60 days, 957 (5.8%) of the patients were still hospitalized. Table S1 in the Additional file 1 shows the number of patients who survived, were discharged alive or died in each time interval in which the follow up had been partitioned.

Demographic and clinical characteristics of the patients are listed in the Additional file 1: Tables S2 and S3. Discharged patients had spent 10.8 days (median) (IQR 6.8–18.6 days) in the ICU, 19.9 days (IQR 12.6– 31.9 days) in the hospital, and had received mechanical ventilation for 7.0 days (median) (IQR 3.8–13.7).

### Protein intake

Records for 142,726 days on diet were available for the analysis (on average, 10.1 days per patient). Including assumptions on protein intake after ICU discharge, a total of 166,976 days were included in the analysis. 12,854 patients (77.9%) did not require imputations for protein intake after ICU discharge. Among patients not requiring those assumptions, 21,771 days (15.3% of days) were identified, on which patients had received parenteral amino acids in addition to, or instead of enteral protein. On average, EN accounted for 85.4% of total protein intake.

On at least one day on diet, 14,859 of 16,489 patients (90.1%) received low amounts of protein (level I, < 0.8 g/kg per day). On 44.0% of the days, patients received less than 0.8 g protein/kg per day (level I); on 32.8% of the days, protein intake was in the range of 0.8–1.2 g protein/kg per day (level II), and on 23.2% of the days, protein intake was above 1.2 g/kg per day (level III).

Based on days with parenteral and/or enteral protein intake, we also calculated the average daily protein intake for the different intake levels (low, standard, high):

Low protein diet (level I, < 0.8 g/kg per day): 0.49 (median) g protein/kg per day [IQR 0.27–0.66]; standard protein diet (level II, 0.8–1.2 g/kg day): 0.99 g/kg per day [IQR 0.89–1.09] and high protein diet (level III, > 1.2 g/kg per day): 1.41 g/kg per day [1.29–1.60].

There was a close correlation (*r* = 0.88) between daily protein and calorie intake (Additional file 1: Figure S2). Referred to the level of protein intake, daily total calorie intake was 11.69 (median) kcal/kg [IQR 6.91–16.48] with level I, 22.47 kcal/kg [19.04–25.71] with level II, and 27.78 kcal/kg [24.00–32.00] with level III.

### Association of protein intake with the hazard of in-hospital death/live hospital discharge

The associations of the variables in the confounder model with the outcome are presented in the Additional file 1: Figures S3 and S4A/B. There was no evidence that the number of days with PN or with oral intake (during the first three days with MNT) was associated with the rate of in-hospital death. Rates of live hospital discharge, however, were lower with a longer duration of PN.

**Fig. 2:**
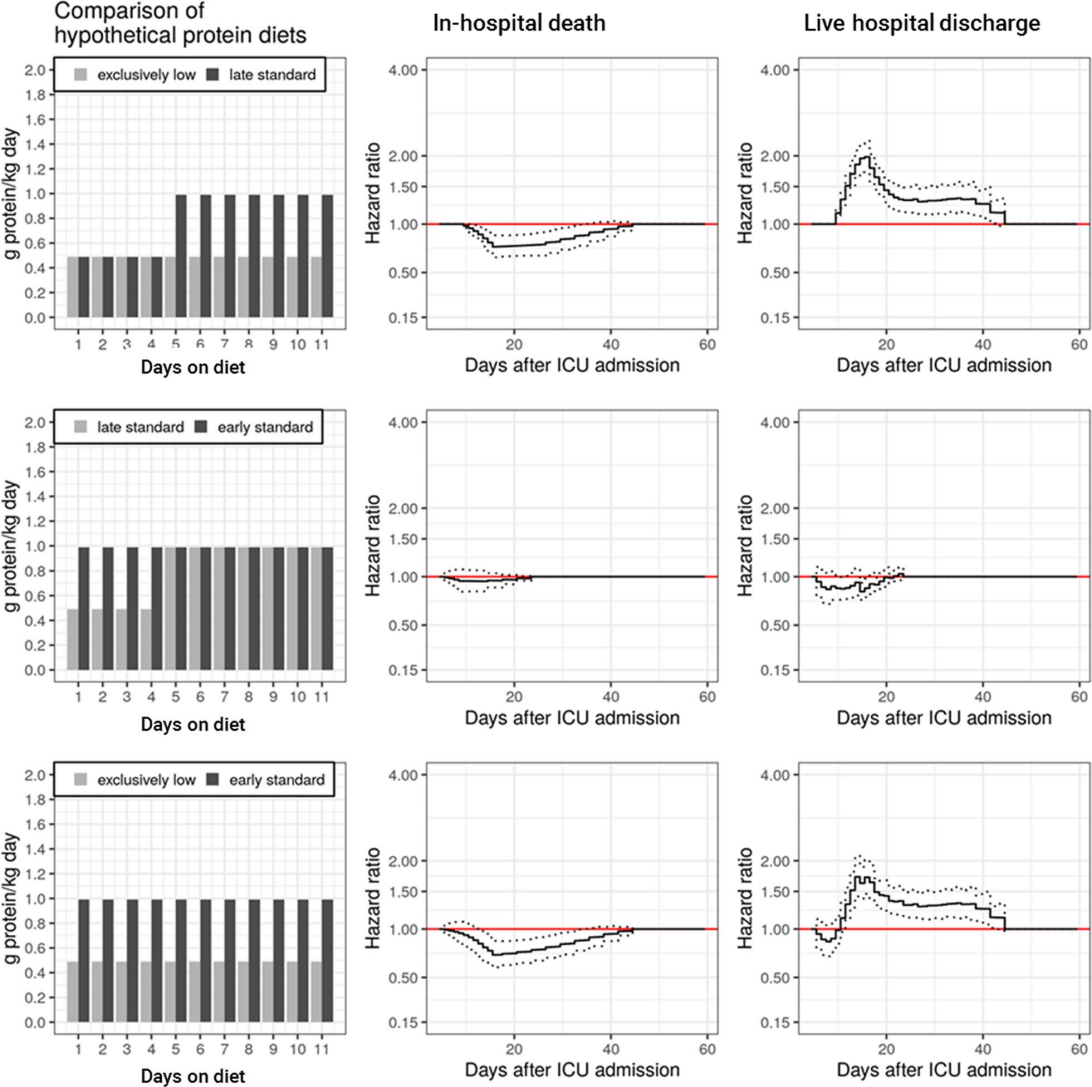
Comparison of an early or late standard protein intake with a low protein intake. Column 1: design of diet comparisons analyzing different hypothetical protein diets (pseudo-observations) (Table 1). Protein intake reflects the median of corresponding categories (standard: 0.8–1.2 g protein/kg per day; low: < 0.8 g/kg per day). Column 2 and 3: corresponding time-varying associations of different hypothetical diets with the hazard of in-hospital death or live hospital discharge (cause-specific hazards). Solid lines indicate hazard ratios (HR), hatched lines indicate corresponding 95% confidence intervals (CI) (HRs and CIs for specific time intervals after ICU admission are presented in the Additional file 1: Tables S4 and S6). Reference diet is that which provides fewer protein (e.g., an HR (and 95% CI) < 1 would indicate that the hazard of in-hospital death/live hospital discharge associated with the diet providing more protein was smaller). Please note that HRs (and corresponding 95% CIs) must be 1 for the first time interval between day 4 and 5 (due to the specification of the lag time), and also for time intervals, in which protein intake of both hypothetical diets is identical within the relevant time window that affects the hazard

**Fig. 3:**
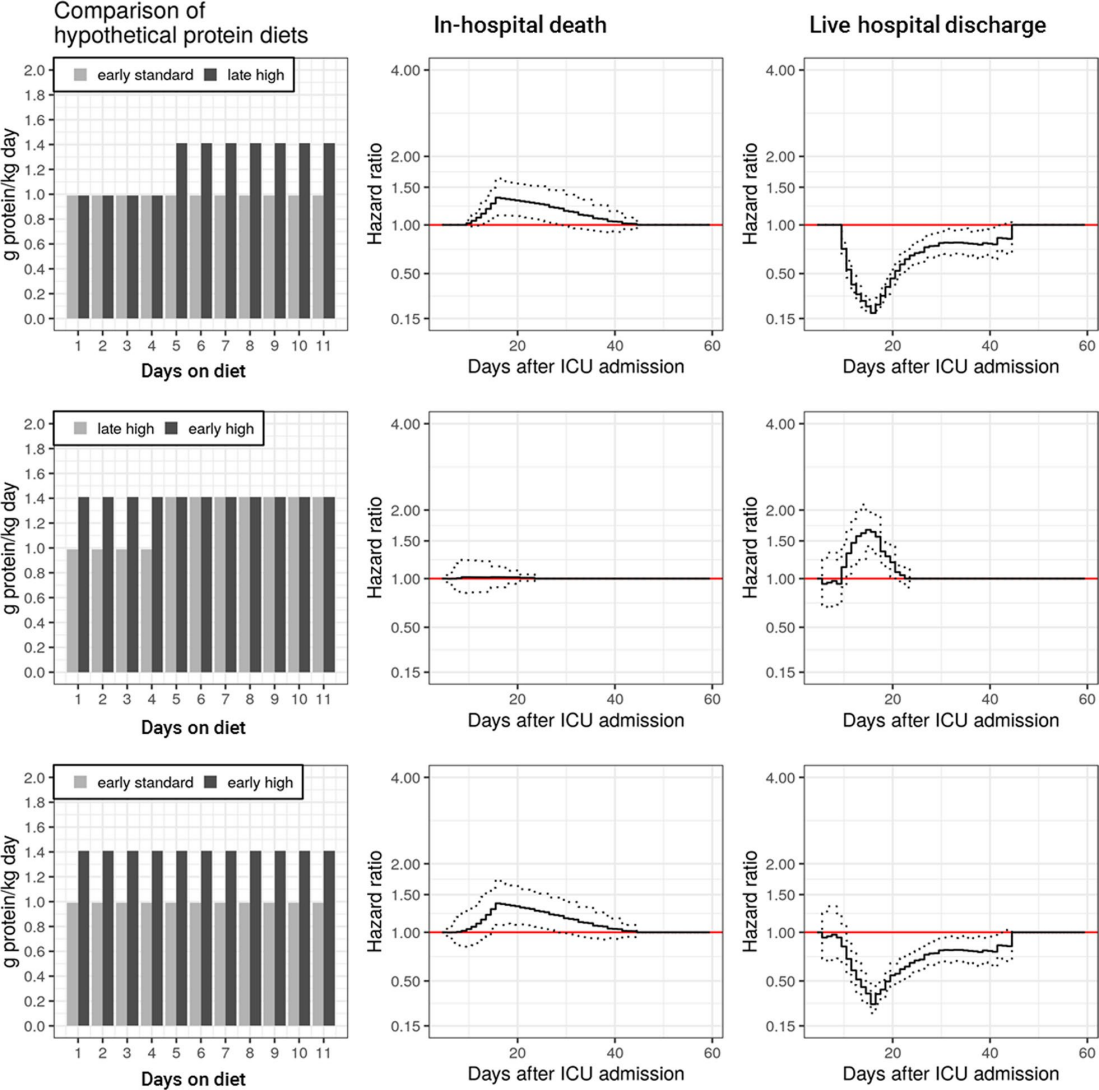
Comparison of an early or late high protein intake with a standard protein intake. Column 1: design of diet comparisons analyzing different hypothetical protein diets (pseudo-observations) (Table 1). Protein intake reflects the median of corresponding categories (standard: 0.8–1.2 g protein/kg per day; high: > 1.2 g/kg per day). Column 2 and 3: corresponding time-varying associations of different hypothetical diets with the rate of in-hospital death or live hospital discharge (cause-specific hazards). Solid lines indicate hazard ratios (HR), hatched lines indicate corresponding 95% confidence intervals (CI) (HRs and CIs for specific time intervals after ICU admission are presented in the Additional file 1: S6 and S7). Reference diet is that which provides fewer protein (e.g., an HR (and 95% CI) < 1 would indicate that rate of in-hospital death/live hospital discharge associated with the diet providing more protein was smaller). Please note that HRs (and corresponding 95% CIs) must be 1 for the first time interval between day 4 and 5 (due to the specification of the lag time), and also for time intervals, in which protein intake of both hypothetical diets is identical within the relevant time window that affects the hazard

We identified several significant associations between daily protein intake and outcome. These associations (hazard ratios) were time-varying (transient), and corresponding hazard ratios reached their maximum/minimum value in the third week after ICU admission. Figures 2 and 3 show the results of the comparisons of different hypothetical protein intakes based on hazard ratios (relative risks). Corresponding absolute risks (cumulative incidence functions) are shown in the Additional file 1: Figures S6 and S7).

#### Comparison of a standard protein intake with a low protein intake (*Fig. **2*, Additional file 1: Tables S4 and S6)

We compared three different hypothetical protein diets:

- an exclusively low protein diet (daily feeding of less than 0.8 g protein/kg (level I) on days on diet #1 to day #11, about 0.49 g protein/kg per day);
- a late standard protein diet (daily feeding of 0.8 to 1.2 g protein/kg (level II) on days #5 to #11, about 0.99 g protein/kg per day).
- an early standard protein diet (daily feeding of 0.8 to 1.2 g protein/kg (level II) on days #1 to #4).

Compared to an exclusively low protein diet, cause-specific model specifications estimated an improved outcome when adding extra protein to an exclusively low protein diet on days #5 to #11 (thereby creating a late standard protein diet) (Fig. 2, upper panel). This diet was associated with a lower rate of in-hospital death (minimum HR 0.75 (95% CI 0.64, 0.87) and a higher rate of live hospital discharge (maximum HR 1.98 (95% CI 1.72, 2.28). In comparison with a late standard protein diet, an early standard protein diet was not associated with improved outcomes (Fig. 2, middle and lower panel).

With regard to in-hospital death, similar results were obtained in a sensitivity analysis. For that analysis, we had analyzed ICU outcomes (instead of hospital outcomes), which did not require assumptions concerning protein intake after ICU discharge (for relative risks, see Additional file 1: Figure S11, for absolute risks Additional file 1: Figure S13). In contrast to in-hospital death, however, results on live hospital discharge could not be reproduced in that sensitivity analysis. Unlike the main analysis, this sensitivity analysis did not consider patients’ outcomes after discharge from the ICU.

#### Comparison of a high protein intake with a standard protein intake (*Fig. **3*, Additional file 1: Tables S5 and S7)

Comparisons of protein diets included two additional hypothetical diets:

- a late high protein diet (daily protein intake of > 1.2 g/kg (level III, about 1.41 g/kg per day) on days #5 to #11)
- an early high protein diet (daily feeding of more than 1.2 g protein/kg (level III) on days #1 to #4).

The main finding of the primary analysis was that, in comparison with an early standard protein diet, an early or late high protein diet was significantly associated with a worse outcome being particularly evident for the hazard of live hospital discharge (minimum HR 0.19 (95% CI 0.15, 0.24, Fig. 2, upper panel). However, according to our sensitivity analysis (ICU outcomes only), these results may be biased by our assumption of a standard protein intake after ICU discharge (for relative risks, see Additional file 1: Figure S12, for absolute risks Additional file 1: Figure S14). Assigning a standard intake to presumably healthier patients may artificially worsen outcomes associated with a high protein intake in sicker patients still being treated on the ICU. Without those assumptions, a high protein intake appeared to neither worsen nor improve ICU outcomes.

All findings of the main, cause-specific hazard analysis (dynamic time window) were qualitatively comparable with the results of the subdistributional hazard analysis (for relative risks, see Additional file 1: Figure S5, for absolute risks Additional file 1: Figure S8; corresponding definitions of hypothetical diets are presented in the Additional file 1: Table S8)., and of a sensitivity analysis, for which we had used a static time window for evaluating outcomes (for relative risks, see Additional file 1: Figures S9 and S10).

### Association of protein intake with the hazard of in-hospital death/live hospital discharge in subgroups

#### Obese patients

At ICU admission, 4277 patients had a BMI > 30 kg/m^2^. Analysis of this subgroup revealed associations between hypothetical diets and in-hospital death/live hospital discharge which were qualitatively comparable to those of the main analysis (for relative risks, see Additional file 1: Figures S15 and S16).

### Patients with a different total calorie intake

On day 3 after ICU admission, 3518 patients had a low calorie intake (< 30% of target, 4.03 kcal/kg per day [IQR 2.50–5.71]), 4569 a moderate intake (30–70% of target, 12.36 kcal/kg per day [IQR 9.47–15.38]), and 8402 a high intake (> 70% of target, 23.10 kcal/kg per day [IQR 19.18–27.40]); analysis of these subgroups revealed that associations between hypothetical protein diets and in-hospital death/live hospital discharge may possibly depend on total calorie intake during the early acute phase (for relative risks of different diet comparisons, see Additional file 1: Figures S17–S22). Associations with outcomes were most prominent for a late standard protein intake analyzed in patients having a low calorie intake on day three after ICU admission. Associations of a high protein intake with outcomes, however, appeared not to depend on early calorie intake.

Limitations of this subgroup analysis result from the comparatively small number of patients having a moderate or high protein intake in combination with a low total calorie intake early after ICU admission. Subgroup-specific associations of a late standard or a late high protein diet with outcomes are less prone to error, and are presented in a comparative way in Additional file 1: Figure S23.

## Discussion

In this large multi-center database analysis, we analyzed associations between a varying, predominantly enteral protein intake during the early and late acute phase of critical illness, and the hazard of in-hospital death or of live hospital discharge. An actual intake of 0.8–1.2 g protein instead of less than 0.8 g protein /kg day during the late acute phase (beyond ICU day 4) was associated with a lower hazard of in-hospital death and a higher hazard of live hospital discharge. Compared to a standard intake, a high protein intake (> 1.2 g/kg day) was not associated with a better outcome, but may possibly worsen prognosis. Similar results were obtained in the subgroup of overweight patients.

Beneficial associations of a standard protein intake with live hospital discharge, however, are uncertain. For surviving patients, who had been discharged from the ICU before day #11, our primary analysis required imputation of a daily protein intake up to day on diet #11. For imputation we used a standard intake of 0.8–1.2 g protein/kg per day. To consider a potential bias by this imputation, we performed a sensitivity analysis using in-ICU death and live ICU discharge as competing risks. Analysis of ICU outcomes did not require assumptions regarding protein intake after ICU discharge. This sensitivity analysis, however, could not reproduce the advantageous association of a standard protein intake with live hospital discharge found by the primary analysis.

This discrepancy may have two explanations: beneficial effects may be an artefact of the main analysis attributing a standard intake to (healthier) patients discharged alive from the ICU before day 11, or advantageous effects may have escaped sensitivity analysis, which could not evaluate benefits of a standard intake on hospital mortality rates after ICU discharge.

Currently, there is no adequately powered randomized multicenter trial on the effect of a standard protein intake and its timing (early vs. late) on outcome of critically ill patients. To fill this knowledge gap, some authors extracted protein intake from large randomized studies originally designed to study the effects of a different calorie intake. The largest meta-analysis by Davies et al. [8] included 14 randomized studies (3238 patients) and found that increasing average daily protein intake by 0.3 g/kg per day (from 0.7 to 1.0 g/kg per day) did not improve morbidity or mortality. A post hoc analysis of the PermiT trial [29] obtained similar results. Likely, in these studies variations of protein intake were too small to produce relevant clinical effects. The meta-analysis by Tian et al. [7], however, showed that an intake < 0.65 g protein/kg ideal body weight per day in the acute phase increased the rate of infection (compared with an intake > 0.85 g protein/kg ideal body weight per day). These results would be compatible with our findings derived from the comparison of an exclusively low protein diet (0.49 g protein/kg per day) with an early or late standard protein diet providing, on average, 0.99 g protein/kg per day on days on diet #1 to #4, or #5 to #11.

Reasons why an early standard protein intake was not associated with an outcome benefit, may relate to the time-dependency of metabolic reactions during critical illness. During the early acute phase, protein metabolism demonstrates a comparably poor responsiveness towards exogenous nutrients. In contrast to healthy individuals, administration of protein is unable to antagonize increased rates of protein catabolism effectively [30]. Later on, during the late acute phase, anabolic resistance may decrease and thus allowing exogenous protein to increasingly spare protein. This effect may explain coherences between a late standard protein diet and a better outcome [31, 32].

Our finding that a high protein intake was not associated with a better outcome is in contrast to the results of the majority of observational and mechanistic studies suggesting that a high protein intake early during ICU stay is beneficial. Elke et al. [4] reviewed observational studies, and found that beneficial associations were quite variable showing linear, non-linear (stepped) or phase- and disease-specific patterns. Only two small studies from the same institution reported a worse 6-month mortality, if patients had been exposed to a high protein intake (> 1.2 g/kg day) during the early acute phase [25, 33]. Numerous analytical and conceptual weaknesses, however, render the interpretation of preceding observational studies almost impossible. Significant limitations result from an inadequate ratio between the number of events and confounding variables, and from the ignorance of confounding by indication, competing risks, time-dependency of protein intake, and time-variation/non-linearity of associations, causing a considerable bias [4, 12, 34, 35].

The recent meta-analysis by Lee et al. [10] analyzed the effect of a high protein intake in 19 randomized studies (1731 patients) and found that increasing average daily protein intake by 0.4 g/kg per day (from 0.9 to 1.3 g/kg per day) did not affect overall mortality or other clinical or patient-centered outcomes. However, most of the included studies were of moderate quality, small and single-center. Several studies were not specifically designed to examine effects of a high protein diet on acute outcomes, and negative effects of too little and too much protein may have cancelled each other out.

The results of our main analysis revealed unfavorable associations between a high protein intake and hospital outcomes, especially with regard to the hazard of live hospital discharge. Due to methodological constraints, however, our analysis can neither prove nor exclude such detrimental effects. The result of our sensitivity analysis on ICU outcomes suggests that assuming a standard protein intake for presumably healthier patients after ICU discharge may have contributed to unfavorable associations of a high protein intake in the main analysis. As noted above, an advantage of the ICU model is, that it doesn’t conflate imputation of protein intake with the patients’ health. On the other hand, this analysis does not model the association of nutrition and hospital outcomes after discharge from ICU. If detrimental effects of a high protein intake had existed after live ICU discharge, our sensitivity analysis would have missed them.

Association of a high (predominantly enteral) protein intake with a worse outcome would not be without scientific basis. Unfavorable findings may result from an intestinal amino acid surplus during the acute phase. Catabolic states may decrease the amount of amino acid absorption and release to the circulation [36, 37]. Together with a high intake, this will cause an intestinal amino acid excess, which may not be harmless. Enteral amino acids are important for growth and function of a possibly pathological microbiome [38], and may suppress intestinal autophagy thereby promoting mucosal inflammation and damage, and impairing local anti-bacterial defense [39, 40]. Various experimental studies revealed that a high protein diet may reduce antifungal activity [41], promote parasitemia [42], or facilitate bacterial translocation [43]. In severely malnourished edematous adults, provision of a high protein diet during the initial phase of treatment resulted in a threefold increase in mortality [44].

### Limitations and strengths

Confounding by indication is a major point of criticism in observational studies examining the importance of enteral nutrient intake for outcomes of critically ill patients (as our study also did). Within the present analysis, confounding may arise from factors that were unobserved or not adjusted for, but still affected the outcome as well as nutrition. These factors also include the decisions that are taken during the courses of ICU to increase or decrease protein intake based on changing patient condition. Thus, healthier patients have a better clinical outcome, and the same forces that make them healthier, cooperate to make them easier to feed via the enteral route.

We used the following strategies to account for this source of confounding: a) by incorporating a lag time of four days between a specific day on diet, and the association of this diet with the subsequent event of interest (in-hospital death, live hospital discharge), we minimized interferences from a better nutritional tolerance prior to discharge, and from a worse tolerance prior to death, thereby eliminating the most immediate confounding by indication, b) by adjusting to the duration of oral and parenteral nutrition during the first four days of ICU stay, we directly accounted for interferences from gastrointestinal function immediately after the insult, being presumably better with oral, and worse with parenteral feeding.

Our results suggest that these strategies were effective: If confounding by indication had been strong, we should have observed significant associations between an increasing protein intake during the early acute phase and a progressively better outcome (which was not the case).

We could not separately analyze the relative contribution of carbohydrate/fat versus protein intake to the associations with different outcomes. Several large randomized studies showed that variations of non-protein calorie intake in the range of 400–600 kcal/day were unimportant for morbidity and mortality of critically ill patients [45, 46]. According to our subgroup analysis, however, we cannot exclude the possibility that some interactions exist. Associations of a late standard protein intake with a better outcome were the strongest in those patients with a low total calorie intake during the early acute phase.

Furthermore, we do not know whether results would have been the same, if our patients had largely received parenteral amino acids instead of enteral protein. In contrast to amino acids from enteral protein, parenteral amino acids are not subject to intestinal digestion, but may still inhibit autophagy [47].

Finally, we can also not exclude some selection bias, resulting from the intrinsic motivation of the investigator (and of her/his ICU) to contribute data to the database. Since, however, patient characteristics of our study (age, BMI, Apache II, hospital mortality) were virtually identical with those reported recently by large PRCTs (PermiT, TARGET) examining effects of a variable nutrient intake on outcome [45, 46], we feel that a generalization of our results is still possible.

The main strength of our study is the large number of patients studied allowing a generalization of the findings, and the methodological approach specifically guided by the inherent analytical problems of observational nutrition studies. By considering competing risks, the time-varying nature of nutrition, and a potential indication bias, we could eliminate some of the major shortcomings of preceding observational studies’ analyses.

## Conclusion

In critically ill patients, increasing protein intake from about 0.5 g protein/kg per day to 1.0 g protein/kg per day on days #5 to #11 after ICU admission was associated with a significantly lower in-hospital mortality and, possibly, also with a shorter time until life hospital discharge. Giving more protein early or late after ICU admission (up to 1.4 g/kg day) was not associated with a further improvement of outcomes. A high protein intake may even prolong time until hospital discharge. This unfavorable result, however, was qualitatively changed by a sensitivity analysis. Although our results are only hypothesis generating, they strongly speak in favor of a late standard protein intake to optimize patient outcomes.

## Supplementary Information

**Additional file 1**: Supplementary information and results.

## Data Availability

Analysis code deposited at time of publication is available at 10.5281/zenodo.5567367. The repository is maintained at https://github.com/adibender/analysis-protein-intake-2021.

## Abbreviations

ICU: Intensive care unit
HR: Hazard ratio
CI: Confidence interval
BMI: Body Mass Index
kg: Kilogram
EN: Enteral nutrition
PN: Parenteral nutrition
MNT: Medical nutrition therapy
MV: Mechanical ventilation
