# Supplementary material for: Protein intake and outcome of critically ill patients: analysis of a large international database using piece-wise exponential additive mixed models
Source: Crit Care. 2022 Jan 11;26:7. doi: 10.1186/s13054-021-03870-5 (PMC8751086; doi:10.1186/s13054-021-03870-5)

Protein intake and outcome of critically ill patients requiring mechanical ventilation – analysis of a large international database using piece-wise exponential additive mixed models (PAMMs)

Additional File

1. Methods

1.1 Preceding publications from the surveys

Data of other, smaller subgroups of the current database have been incorporated into the following publications:

1. Alberda C, Gramlich L, Jones N, Jeejeebhoy K, Day AG, Dhaliwal R, Heyland DK. The relationship between nutritional intake and clinical outcomes in critically ill patients: results of an international multicenter observational study. Intensive Care Med. 2009 Oct;35(10):1728-37.
2. Heyland DK, Cahill NE, Dhaliwal R, Sun X, Day AG, McClave SA. Impact of enteral feeding protocols on enteral nutrition delivery: results of a multicenter observational study. JPEN J Parenter Enteral Nutr. 2010 Nov-Dec;34(6):675-84.
3. Heyland DK, Cahill N, Day AG. Optimal amount of calories for critically ill patients: depends on how you slice the cake! Crit Care Med. 2011 Dec;39(12):2619-26.
4. Heyland DK, Stephens KE, Day AG, McClave SA. The success of enteral nutrition and ICU-acquired infections: a multicenter observational study. Clin Nutr. 2011 Apr;30(2):148-55.
5. Martino JL, Stapleton RD, Wang M, Day AG, Cahill NE, Dixon AE, Suratt BT, Heyland DK. Extreme obesity and outcomes in critically ill patients. Chest. 2011 Nov;140(5):1198-206.
6. Kutsogiannis J, Alberda C, Gramlich L, Cahill NE, Wang M, Day AG, Dhaliwal R, Heyland DK. Full use of supplemental parenteral nutrition in critically ill patients: results of an international multicenter observational study. Crit Care Med. 2011 Dec;39(12):2691-9.
7. Elke G, Wang M, Weiler N, Day AG, Heyland DK. Close to recommended caloric and protein intake by enteral nutrition is associated with better clinical outcome of critically ill septic patients: secondary analysis of a large international nutrition database. Crit Care. 2014 Feb 10;18(1):R29
8. Nicolo M, Heyland DK, Chittams J, Sammarco T, Compher C. Clinical Outcomes Related to Protein Delivery in a Critically Ill Population: A Multicenter, Multinational Observation Study. JPEN J Parenter Enteral Nutr. 2016 Jan;40(1):45-51.
9. Compher C, Chittams J, Sammarco T, Higashibeppu N, Higashiguchi T, Heyland DK. Greater Nutrient Intake Is Associated With Lower Mortality in Western and Eastern Critically Ill Patients With Low BMI: A Multicenter, Multinational Observational Study. JPEN J Parenter Enteral Nutr. 2019 Jan;43(1):63-69.
10. Hartl WH, Bender A, Scheipl F, Kuppinger D, Day AG, Küchenhoff H. Calorie intake and short-term survival of critically ill patients. Clin Nutr. 2019 Apr;38(2):660-667.
11. Chourdakis M, Grammatikopoulou MG, Day AG, Bouras E, Heyland DK. Are all low-NUTRIC-score patients the same? Analysis of a multi-center observational study to determine the relationship between nutrition intake and outcome. Clin Nutr. 2019 Dec;38(6):2783-2789.
12. Iwuchukwu C, O'Keefe GE, Day AG, Jiang X, Heyland DK. Application of the modified Nutrition Risk in Critically Ill score to nutritional risk stratification of trauma victims: A multicenter observational study. J Trauma Acute Care Surg. 2020 Dec;89(6):1143-1148.
13. Heyland DK, Ortiz A, Stoppe C, Patel JJ, Yeh DD, Dukes G, Chen YJ, Almansa C, Day AG. Incidence, Risk Factors, and Clinical Consequence of Enteral Feeding Intolerance in the Mechanically Ventilated Critically Ill: An Analysis of a Multicenter, Multiyear Database. Crit Care Med. 2021 Jan 1;49(1):49-59.
    1. Statistical analysis

1.2.1 Concept

We used both a cause-specific and a subdistributional competing risk model, where the hazard was a piecewise constant function depending on the protein intake and confounder variables. The confounder model was defined based on substantive and methodological considerations, resulting in a flexible nonlinear model with partially time-varying and partially time-constant associations. Nonlinear associations were estimated using penalized splines (see Bender et al., 2018 for methodological details).

Furthermore, we included a Gaussian random frailty effect for the ICUs in the model to account for heterogeneity of the different ICUs. We included the number of days with propofol therapy, number of days with parenteral or oral nutrition, and the number of days with mechanical ventilation up to day 4 after ICU admission as time-constant linear covariates in the confounder model. For Apache II score we fit linear time-varying associations, whereas age and BMI were modeled smoothly and time-varying.

Subsequently, we modeled the association of protein intake with the risk of death/chance of discharge by adding those variables to the final confounder model. To assess the potentially time-varying association between cumulative protein intake and the outcomes, we used a dynamic time window (time lag of 4 days (*lag-time*), and lead-time = 4 + twice the number of days on diet, Figure S1). This modeling strategy helps to avoid endogeneity (confounding by indication) and accounts for treatment time required before associations of protein intake with outcome might become effective, and for time intervals beyond which such associations are likely to vanish. A static time window was used for a sensitivity analysis (see below).

Figure S1:

Dynamic time window for outcome analysis (for the definition of time intervals see 1.2.2.)


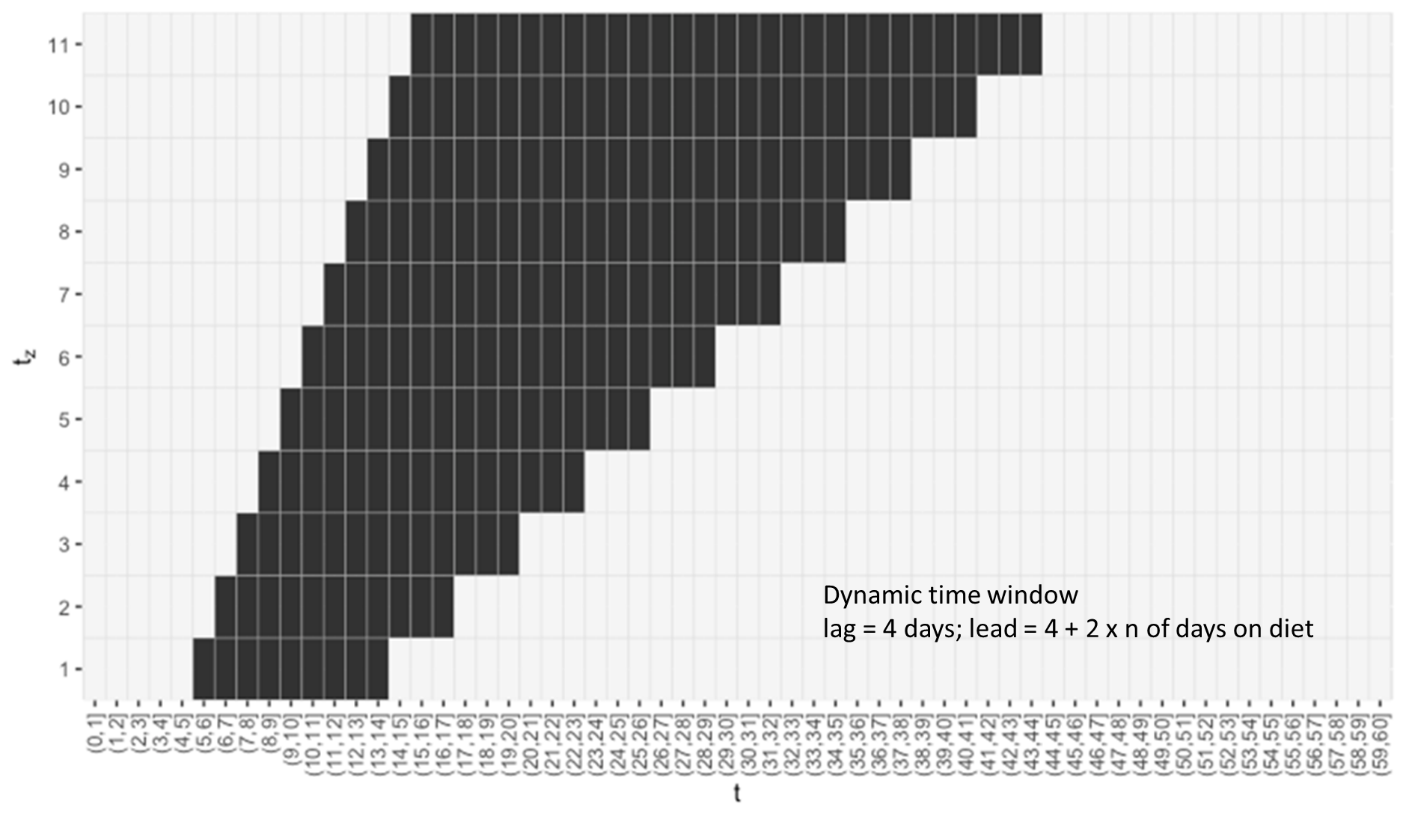


For the modelling of the protein intake, a low protein diet (level I, < 0.8 g/kg per day) was defined as the reference diet. Associations of a higher intake (level II, 0.8 – 1.2 g protein/kg per day. and level III, > 1.2 g protein/kg per day) with outcome were modelled as bivariate smooth functions (smoothly time-varying effects of time-dependent covariates) that depict partial associations between protein intake on days on diet #1 to #11, and the rate of in-hospital death/live hospital discharge (see Bender et al., 2018 for methodological details).

To assess the overall association of protein intake with the outcomes for a specific interval of the follow up, these partial associations of level II or III were added up. The specific partial associations that were cumulated for each interval of the follow-up are delimited by the lag-time of four days and the dynamic lead-time. For example, the estimated rate of in-hospital death/live hospital discharge on day 13 after ICU admission is associated only with the partial effects of days on diet #1 to #9; on the other hand, associations between protein intake on days on diet #1 to #9 with outcome will be only evaluated up to day 2 x 9 + 4= 22 after ICU admission.

## 1.2.2. Interval structure and lag/lead specification

For the implementation of the method described in Bender et al., 2018 (Bender A, Groll A, Scheipl F. A generalized additive model approach to time-to-event analysis. Statistical Modelling. 2018 March 1;18(3–4): 299–321), we discretized the time-scale into days (24h periods) after ICU admission (see Table S1).

Since we only analyzed patients who survived at least 96 hours, our data contains time intervals ranging maximally from (4, 5] to (59, 60] days after ICU admission, i.e. 56 intervals. Table S1 shows the intervals as well as the respective frequencies of events (rates of in-hospital death/live hospital discharge).

The following aspects were important for the complex modeling of the association between protein intake and the hazard rate: a) the model should allow the association between a certain amount of protein intake and the outcome to be cumulative over the relevant period of exposure (see below), and should also potentially be time-varying. b) we assumed that protein intake on a certain day on diet t_e_ can only be associated with individual hazard rates after a certain lag-time (t_lag_). E.g., protein intake on nutrition day $t_{e}$ is associated with the rate of in-hospital death/live hospital discharge within a certain time window during the follow up, and is not associated with that rate outside that time window (Figure S1).

Table S1:

Number of patients, who survived, were discharged alive or died in each time interval in which the follow up had been partitioned.

| interval | at risk (beginning) | dying | discharged alive |
| --- | --- | --- | --- |
| (4,5] | 16489 | 285 | 189 |
| (5,6] | 16015 | 262 | 220 |
| (6,7] | 15533 | 223 | 272 |
| (7,8] | 15038 | 259 | 359 |
| (8,9] | 14420 | 208 | 389 |
| (9,10] | 13823 | 262 | 404 |
| (10,11] | 13157 | 205 | 392 |
| (11,12] | 12560 | 178 | 422 |
| (12,13] | 11960 | 149 | 418 |
| (13,14] | 11393 | 134 | 466 |
| (14,15] | 10793 | 144 | 423 |
| (15,16] | 10226 | 136 | 351 |
| (16,17] | 9739 | 116 | 367 |
| (17,18] | 9256 | 101 | 350 |
| (18,19] | 8805 | 90 | 374 |
| (19,20] | 8341 | 85 | 312 |
| (20,21] | 7944 | 86 | 284 |
| (21,22] | 7574 | 76 | 301 |
| (22,23] | 7197 | 69 | 284 |
| (23,24] | 6844 | 65 | 249 |
| (24,25] | 6530 | 54 | 276 |
| (25,26] | 6200 | 54 | 231 |
| (26,27] | 5915 | 75 | 211 |
| (27,28] | 5629 | 50 | 228 |
| (28,29] | 5351 | 41 | 218 |
| (29,30] | 5092 | 40 | 188 |
| (30,31] | 4864 | 40 | 185 |
| (31,32] | 4639 | 42 | 185 |
| (32,33] | 4412 | 38 | 164 |
| (33,34] | 4210 | 38 | 167 |
| (34,35] | 4005 | 36 | 153 |
| (35,36] | 3816 | 29 | 150 |
| (36,37] | 3637 | 42 | 136 |
| (37,38] | 3459 | 39 | 136 |
| (38,39] | 3284 | 37 | 110 |
| (39,40] | 3137 | 28 | 133 |
| (40,41] | 2976 | 34 | 111 |
| (41,42] | 2831 | 22 | 131 |
| (42,43] | 2678 | 41 | 105 |
| (43,44] | 2532 | 22 | 116 |
| (44,45] | 2394 | 17 | 87 |
| (45,46] | 2290 | 23 | 94 |
| (46,47] | 2173 | 15 | 86 |
| (47,48] | 2072 | 18 | 96 |
| (48,49] | 1958 | 19 | 83 |
| (49,50] | 1856 | 22 | 86 |
| (50,51] | 1748 | 25 | 76 |
| (51,52] | 1647 | 14 | 72 |
| (52,53] | 1561 | 9 | 61 |
| (53,54] | 1491 | 15 | 67 |
| (54,55] | 1409 | 14 | 68 |
| (55,56] | 1327 | 13 | 84 |
| (56,57] | 1230 | 7 | 70 |
| (57,58] | 1153 | 15 | 67 |
| (58,59] | 1071 | 14 | 50 |
| (59,60] | 1007 | 11 | 39 |

## 1.2.3. Model specification

The full model specification for our analysis is given below.

Let $t:=\left( \kappa_{j}-\kappa_{j-1} \right)/2$ the interval-midpoints for intervals $j=1,\ldots,J$. We model the log-hazard rate for patient *i* = 1,…,n as

$$log\left( \lambda_{k,i}\left( tx \right) \right)=\lambda_{0k}\left( t \right)+{x'}_{i}\beta+\beta_{Apache}x_{i,Apache}+\beta_{Apache:t}\left( x_{i,Apache}*t \right)+$$

$$f_{age}\left( x_{i,Age} \right)*t+f_{BMI}\left( x_{i,BMI} \right)*t+\sum_{t_{e}} g_{c_{II}}(t_{e}, t)+\sum_{t_{e}} g_{c_{III}}(t_{e}, t)+b_{l_{i}}$$

where

- $\lambda_{0k}\left( t \right)$ represents the piecewise constant baseline hazard for the $k$-th risk (i.e. death and discharge, respectively).
- $X_{i}\beta$ represents all time-constant effects of time-constant covariates including dummy coded variables, year of admission, diagnosis, admis­sion category, gender and number of days patients had received propofol, parenteral nutrition, or oral nutrition, and had required mechanical ventilation during the first three days in the ICU, respectively
- $\beta_{Apache}x_{i,Apache}+\beta_{Apache:t}\left( x_{i,Apache}*t \right)$ is a linear, time-varying effect of the Apache II Score measured at admission to the ICU,
- $f_{age}\left( x_{i,Age} \right)*t$ and $f_{BMI}\left( x_{i,BMI} \right)*t$ are smooth, time-varying effects of age and BMI, modeled as so-called varying coefficients,
- $g_{C_{II}}$ and$g_{C_{III}}$ are lagged smoothly time-varying cumulative effects of protein intake
- $b_{l_{i}}$ is an independent, identically distributed, Gaussian random intercept term attributed to different ICUs in the data set.

1.2.4. Sensitivity analyses

To analyse the hazard rate of in-hospital death/live hospital discharge and its association with protein intake in the primary analysis, certain assumptions had to be made. To check these assumptions, we performed the following sensitivity analyses:

1. Instead of a dynamic time window we used a static time window (static lag-lead time: 4 and 60 days, respectively, Figure S1) assuming alternatively that associations between protein intake and outcome did not depend on the duration of medical nutrition therapy.
2. To consider a potential bias by assuming a daily standard protein intake for those patients who had been discharged alive from the ICU before day 11 after ICU admission, we used in-ICU death and live ICU discharge as competing risks. Analysis of these outcomes did not require assumption regarding protein intake after ICU discharge.

1.2.5. Extended primary analysis

a) Subdistribution proportional hazards models were created allowing us to examine the power of different diets to predict the rate of in-hospital death.

b) To analyze absolute risks we estimated cumulative incidence functions by modeling the cause-specific hazard function of all cases.

1. Results

2.1. Baseline variables

Table S2:

Demographic and clinical patient characteristics (categorical variables); the duration/number of days were calculated up to the third day on diet.

|  | | n | Percent | 60-day  Hospital  mortality (%) |
| --- | --- | --- | --- | --- |
| Year | 2007 | 2220 | 13.5 | 27.6 |
|  | 2008 | 2270 | 13.8 | 26.7 |
|  | 2009 | 2455 | 14.9 | 24.4 |
|  | 2011 | 3363 | 20.4 | 24.1 |
|  | 2013 | 3137 | 19.0 | 25.9 |
|  | 2014 | 3044 | 18.5 | 24.8 |
| Sex | Female | 6377 | 38.7 | 26.2 |
|  | Male | 10112 | 61.3 | 24.9 |
| Duration of mechanical ventilation | 1 - 24 h | 622 | 3.8 | 6.3 |
|  | 25 - 48 h | 731 | 4.4 | 9.6 |
|  | > 48 h | 15136 | 91.8 | 27.0 |
| Number of days with oral intake | 0 | 15429 | 93.6 | 25.6 |
|  | 1 | 640 | 3.9 | 24.2 |
|  | 2 | 308 | 1.9 | 24.4 |
|  | 3 | 112 | 0.7 | 16.1 |
| Number of days with propofol therapy | 0 | 9702 | 58.8 | 25.7 |
|  | 1 | 1676 | 10.2 | 24.9 |
|  | 2 | 1836 | 11.1 | 25.6 |
|  | 3 | 3275 | 19.9 | 24.2 |
| Number of days with parenteral nutrition | 0 | 13846 | 84.0 | 25.4 |
|  | 1 | 474 | 2.9 | 25.7 |
|  | 2 | 680 | 4.2 | 25.4 |
|  | 3 | 1489 | 9.0 | 25.5 |
| Number of days with enteral nutrition | 0 | 3132 | 19.0 | 25.3 |
|  | 1 | 1698 | 10.3 | 25.2 |
|  | 2 | 3455 | 21.0 | 25.8 |
|  | 3 | 8204 | 49.8 | 25.4 |
| Admission category | Surgical /Elective | 1850 | 11.2 | 28.9 |
|  | Medical | 10485 | 63.6 | 17.5 |
|  | Surgical /Emergency | 4154 | 25.2 | 20.1 |
| Admission diagnosis | Gastrointestinal | 2052 | 12.4 | 25.0 |
|  | Cardio-Vascular | 2514 | 15.2 | 29.1 |
|  | Other | 2102 | 12.7 | 23.5 |
|  | Metabolic | 316 | 1.9 | 13.9 |
|  | Neurologic | 2085 | 12.6 | 24.9 |
|  | Orthopedic/ Trauma | 1954 | 11.9 | 14.3 |
|  | Renal | 127 | 0.8 | 35.4 |
|  | Respiratory | 3677 | 22.3 | 27.6 |
|  | Sepsis | 1662 | 10.1 | 33.1 |

Table S3:

Demographic and clinical characteristics (quantitative variables) of the study participants.

|  | Age (years) | Apache II Score | Body mass index  (kg/m^2^) |
| --- | --- | --- | --- |
| Min. | 18.0 | 0.0 | 13.1 |
| 1^st^ Qu. | 48.0 | 17.0 | 22.6 |
| Median | 62.0 | 22.0 | 25.7 |
| Mean | 59.7 | 22.2 | 27.3 |
| 3^rd^ Qu. | 73.0 | 27.0 | 30.2 |
| Max. | 102.0 | 71.0 | 108.5 |

2.2. Correlation between daily protein and calorie intake

Figure S2:


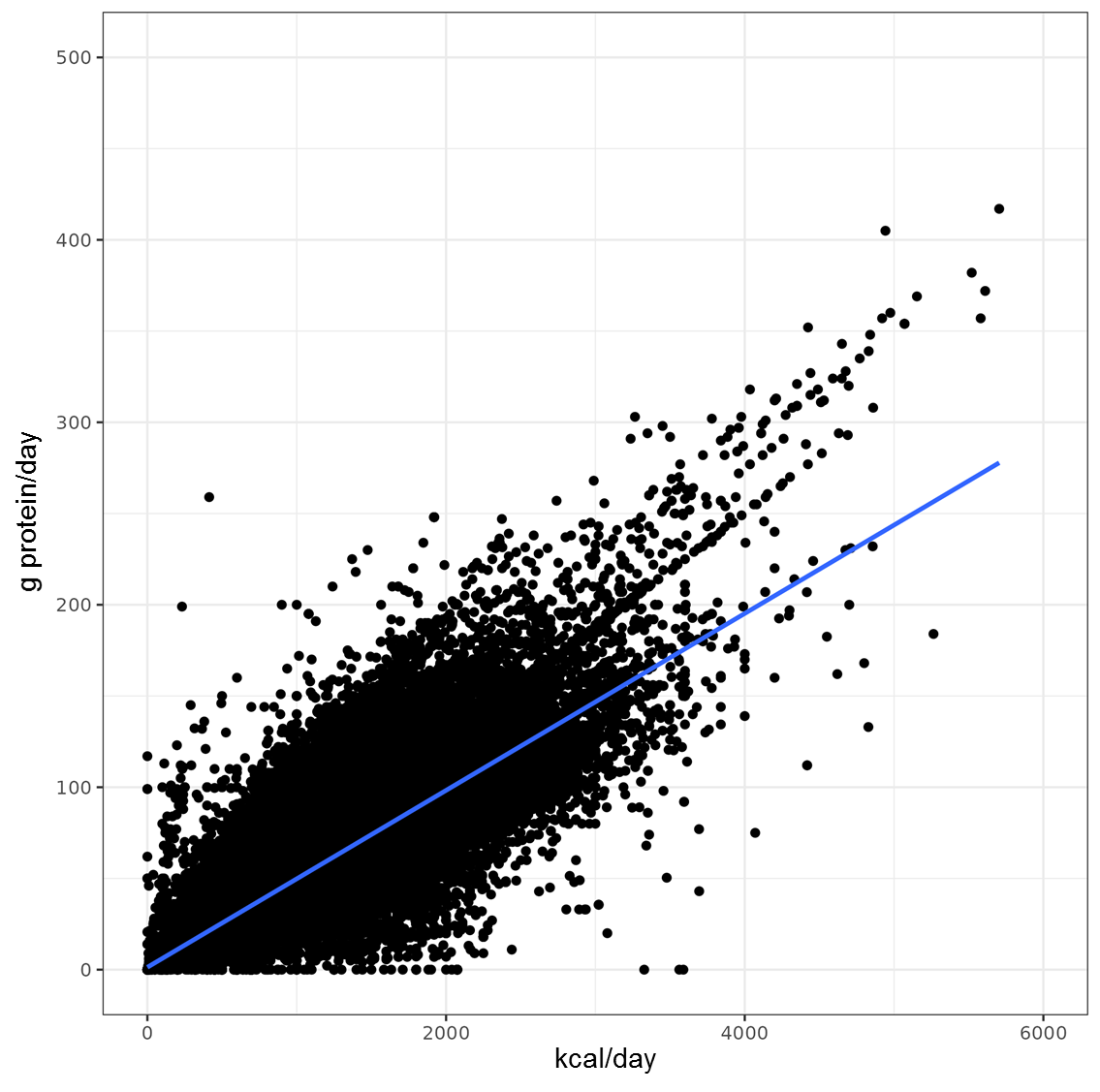


2.3. Association of confounder variables with the rate of in-hospital death/live hospital discharge

Figure S3:

Ln of cause-specific hazard ratios (HR) ($\hat{\beta}$ and 95% confidence intervals) for time-constant covariates with time-constant associations. Horizontal lines represent associations with the ln HR of in-hospital death/live hospital discharge found in the final model including protein covariates. Categorical variables are treatment-coded; the following reference categories were used: Year: 2007, Diagnosis: Other, Admission: Medical, Gender: Female. Variables representing the number (#) of days were included numerically and can take values from 0 to 3 (MV, mechanical ventilation; PN, parenteral nutrition).


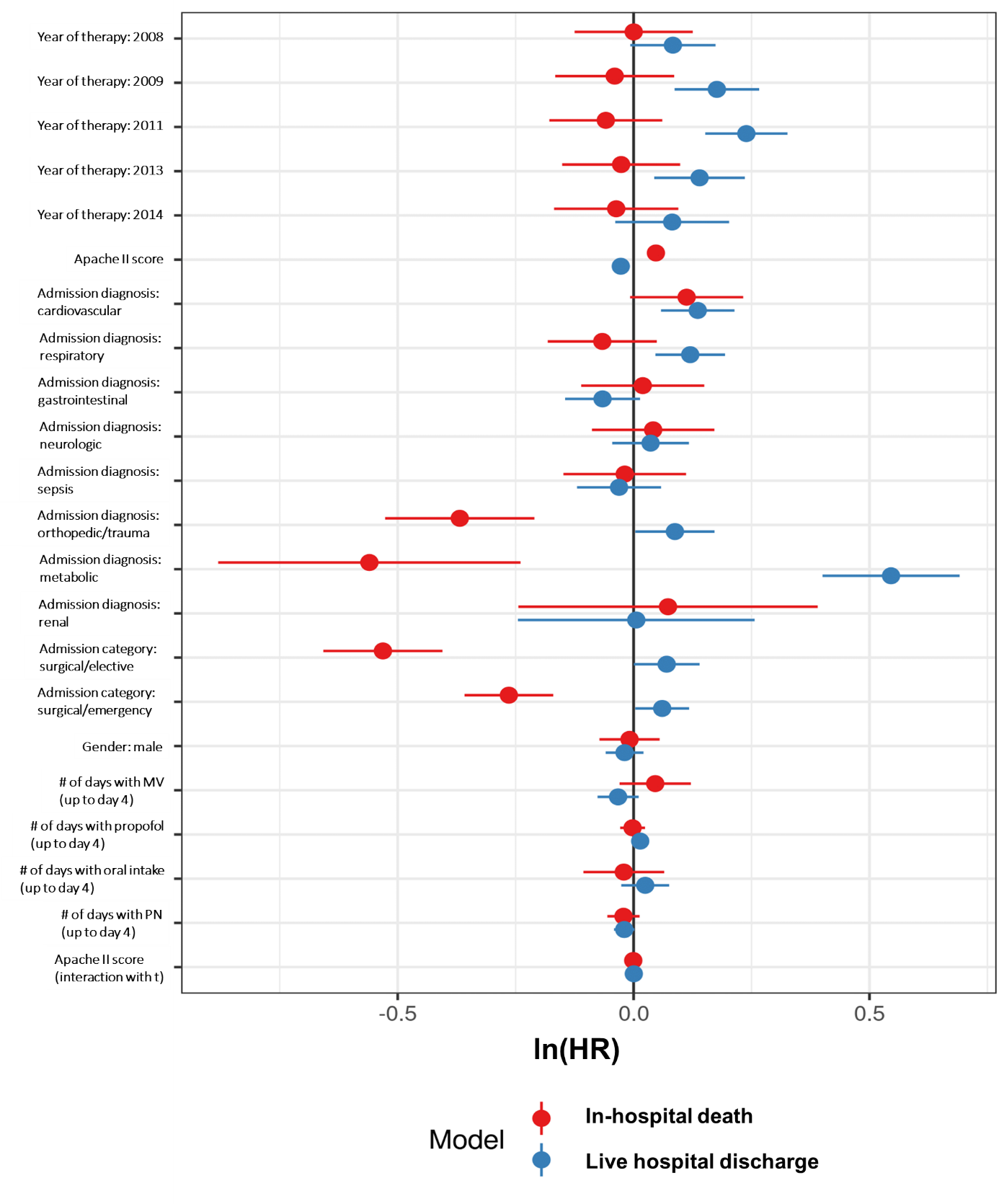


Figures S4A and 4B:

Clockwise: Estimated baseline ln-hazard rate and associations (cause-specific hazard ratios) of age, study center (ICU random effects), and BMI with the rate of in-hospital death (A) and live hospital discharge (B). Dotted lines indicate 95% confidence Intervals (pointwise) of the full model including protein covariates. The Quantile-Quantile plot on the bottom right displays the estimated random effects (frailty) for individual ICUs; these effects vary mostly between -0.25 and 0.25 (there are only a few larger deviations from the theoretical quantiles). Overall, the empirical quantiles are consistent with the assumed Gaussian distribution of the random effects.

1. In-hospital death


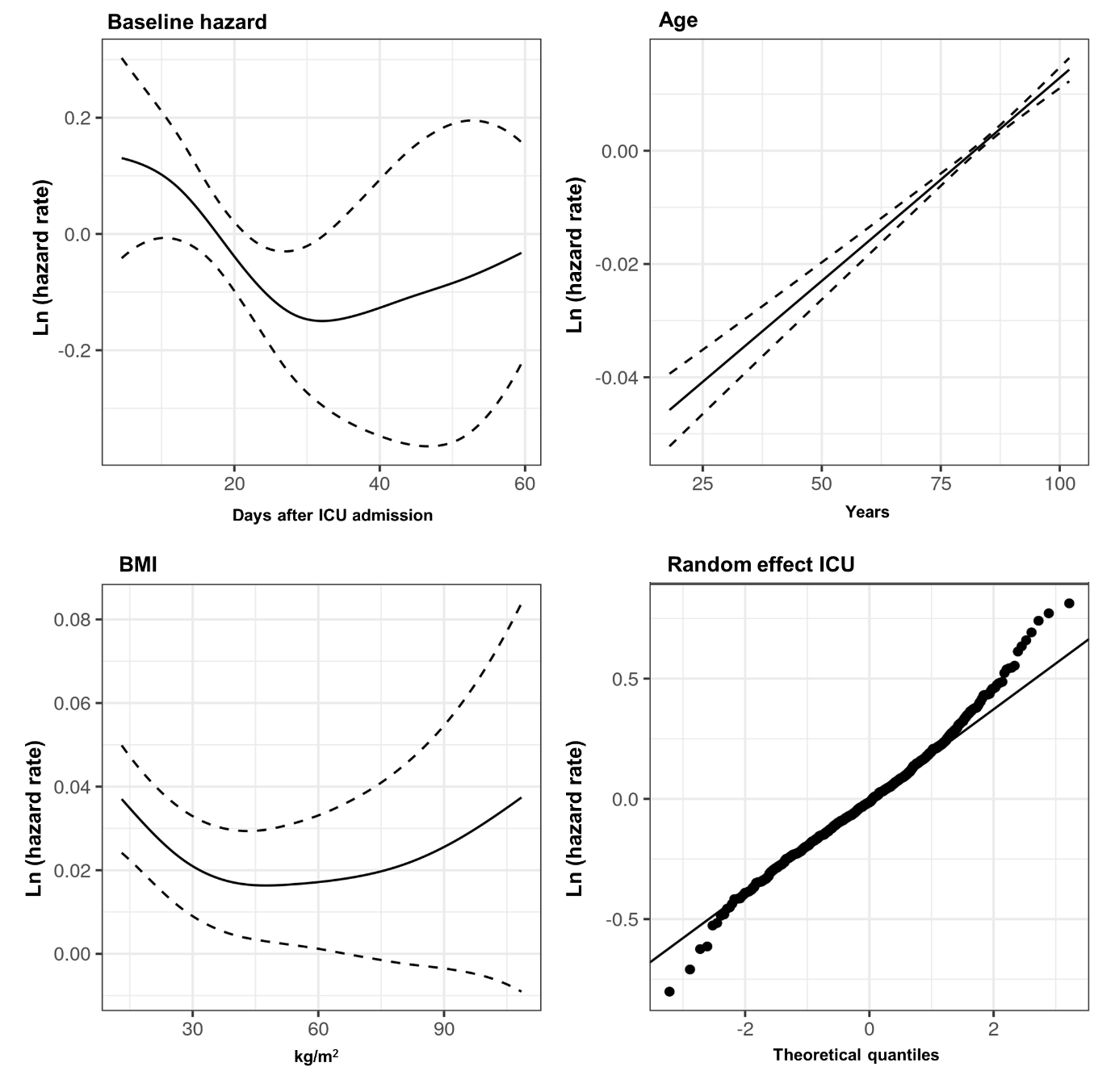


1. Live hospital discharge


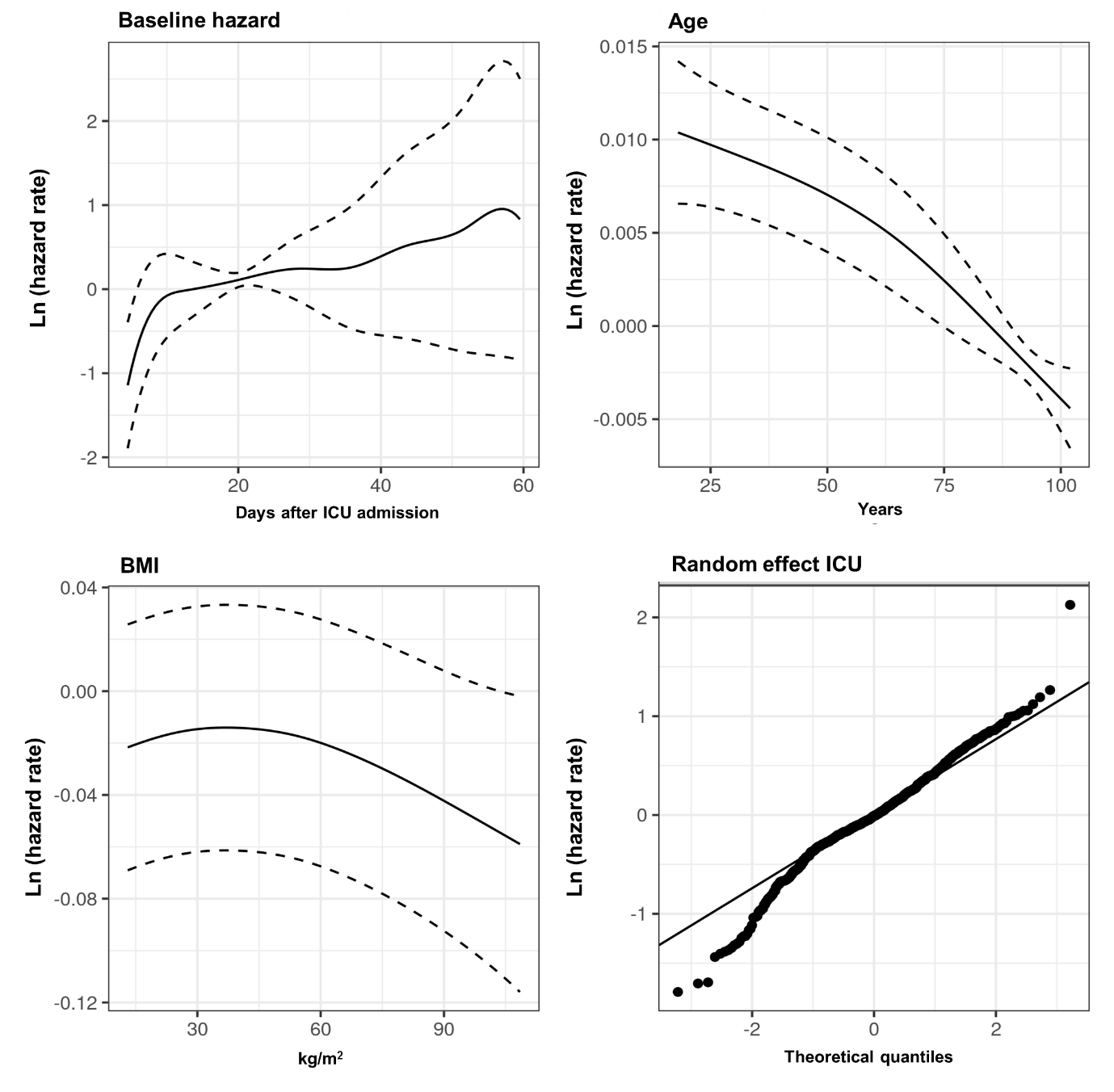


2.4. Association of protein intake with the rate of in-hospital death (cause-specific hazards model)

Table S4: Low versus standard protein intake

Estimated hazard ratios (HR) and corresponding pointwise 95% confidence intervals (CI) for comparisons of protein diets by specific time intervals of the follow up (see also Figures 1 of the main document). Two different rates of protein intake were analyzed: < 0.8 g protein/kg per day (low protein intake) and 0.8 – 1.2 g protein/kg per day (standard protein intake). Three different protein diets were compared: #1: low protein intake on days on diet #1 to #11 (exclusively low protein diet); #2: standard protein intake on days on diet #5 to #11 (late standard protein diet); #3: standard protein intake on days on diet #1 to #4 (early standard protein diet).

|  | Exclusively low protein vs. early standard protein | | Exclusively low protein vs. late standard protein | | Late standard protein vs. early standard protein | |
| --- | --- | --- | --- | --- | --- | --- |
| Interval | HR | CI | HR | CI | HR | CI |
| (4,5] | 1.00 | [1.00, 1.00] | 1.00 | [1.00, 1.00] | 1.00 | [1.00, 1.00] |
| (5,6] | 0.99 | [0.94, 1.04] | 1.00 | [1.00, 1.00] | 0.99 | [0.94, 1.04] |
| (6,7] | 0.98 | [0.9, 1.07] | 1.00 | [1.00, 1.00] | 0.98 | [0.90, 1.07] |
| (7,8] | 0.97 | [0.86, 1.08] | 1.00 | [1.00, 1.00] | 0.97 | [0.86, 1.08] |
| (8,9] | 0.95 | [0.83, 1.09] | 1.00 | [1.00, 1.00] | 0.95 | [0.83, 1.09] |
| (9,10] | 0.93 | [0.80, 1.08] | 0.98 | [0.95, 1.00] | 0.95 | [0.83, 1.08] |
| (10,11] | 0.90 | [0.77, 1.06] | 0.95 | [0.91, 1.00] | 0.95 | [0.83, 1.08] |
| (11,12] | 0.87 | [0.74, 1.03] | 0.92 | [0.86, 0.99] | 0.95 | [0.83, 1.08] |
| (12,13] | 0.83 | [0.70, 1.00] | 0.88 | [0.80, 0.96] | 0.95 | [0.83, 1.07] |
| (13,14] | 0.79 | [0.66, 0.95] | 0.84 | [0.75, 0.93] | 0.95 | [0.83, 1.07] |
| (14,15] | 0.76 | [0.64, 0.90] | 0.79 | [0.70, 0.90] | 0.96 | [0.88, 1.04] |
| (15,16] | 0.71 | [0.59, 0.86] | 0.75 | [0.64, 0.87] | 0.96 | [0.88, 1.04] |
| (16,17] | 0.72 | [0.59, 0.86] | 0.75 | [0.65, 0.87] | 0.95 | [0.87, 1.04] |
| (17,18] | 0.73 | [0.61, 0.86] | 0.75 | [0.65, 0.87] | 0.97 | [0.91, 1.02] |
| (18,19] | 0.73 | [0.61, 0.86] | 0.75 | [0.65, 0.87] | 0.97 | [0.91, 1.02] |
| (19,20] | 0.73 | [0.62, 0.87] | 0.76 | [0.66, 0.87] | 0.96 | [0.91, 1.02] |
| (20,21] | 0.74 | [0.64, 0.87] | 0.76 | [0.66, 0.88] | 0.98 | [0.95, 1.01] |
| (21,22] | 0.75 | [0.64, 0.88] | 0.76 | [0.66, 0.88] | 0.98 | [0.95, 1.01] |
| (22,23] | 0.75 | [0.64, 0.88] | 0.76 | [0.66, 0.89] | 0.98 | [0.95, 1.01] |
| (23,24] | 0.77 | [0.66, 0.89] | 0.77 | [0.66, 0.89] | 1.00 | [1.00, 1.00] |
| (24,25] | 0.77 | [0.66, 0.90] | 0.77 | [0.66, 0.90] | 1.00 | [1.00, 1.00] |
| (25,26] | 0.77 | [0.66, 0.91] | 0.77 | [0.66, 0.91] | 1.00 | [1.00, 1.00] |
| (26,27] | 0.79 | [0.69, 0.92] | 0.79 | [0.69, 0.92] | 1.00 | [1.00, 1.00] |
| (27,28] | 0.80 | [0.69, 0.93] | 0.80 | [0.69, 0.93] | 1.00 | [1.00, 1.00] |
| (28,29] | 0.80 | [0.68, 0.94] | 0.80 | [0.68, 0.94] | 1.00 | [1.00, 1.00] |
| (29,30] | 0.83 | [0.72, 0.95] | 0.83 | [0.72, 0.95] | 1.00 | [1.00, 1.00] |
| (30,31] | 0.83 | [0.72, 0.96] | 0.83 | [0.72, 0.96] | 1.00 | [1.00, 1.00] |
| (31,32] | 0.83 | [0.72, 0.97] | 0.83 | [0.72, 0.97] | 1.00 | [1.00, 1.00] |
| (32, 33] | 0.87 | [0.76, 0.99] | 0.87 | [0.76, 0.99] | 1.00 | [1.00, 1.00] |
| (33,34] | 0.87 | [0.76, 0.99] | 0.87 | [0.76, 0.99] | 1.00 | [1.00, 1.00] |
| (34,35] | 0.87 | [0.76, 1,00] | 0.87 | [0.76, 1.00] | 1.00 | [1.00, 1.00] |
| (35,36] | 0.90 | [0.81, 1.01] | 0.90 | [0.81, 1.01] | 1.00 | [1.00, 1.00] |
| (36,37] | 0.91 | [0.81, 1.02] | 0.91 | [0.81, 1.02] | 1.00 | [1.00, 1.00] |
| (37,38] | 0.91 | [0.81, 1.03] | 0.91 | [0.81, 1.03] | 1.00 | [1.00, 1.00] |
| (38,39] | 0.94 | [0.86, 1.03] | 0.94 | [0.86, 1.03] | 1.00 | [1.00, 1.00] |
| (38,40] | 0.94 | [0.86, 1.03] | 0.94 | [0.86, 1.03] | 1.00 | [1.00, 1.00] |
| (40,41] | 0.94 | [0.86, 1.04] | 0.94 | [0.86, 1.04] | 1.00 | [1.00, 1.00] |
| (41,42] | 0.97 | [0.92, 1.02] | 0.97 | [0.92, 1.02] | 1.00 | [1.00, 1.00] |
| (42,43] | 0.97 | [0.92, 1.03] | 0.97 | [0.92, 1.03] | 1.00 | [1.00, 1.00] |
| (43,44] | 0.97 | [0.92, 1.03] | 0.97 | [0.92, 1.03] | 1.00 | [1.00, 1.00] |
| (44,45] | 1.00 | [1.00, 1.00] | 1.00 | [1.00, 1.00] | 1.00 | [1.00, 1.00] |
| (45,46] | 1.00 | [1.00, 1.00] | 1.00 | [1.00, 1.00] | 1.00 | [1.00, 1.00] |
| (46,47] | 1.00 | [1.00, 1.00] | 1.00 | [1.00, 1.00] | 1.00 | [1.00, 1.00] |
| (47,48] | 1.00 | [1.00, 1.00] | 1.00 | [1.00, 1.00] | 1.00 | [1.00, 1.00] |
| (48,49] | 1.00 | [1.00, 1.00] | 1.00 | [1.00, 1.00] | 1.00 | [1.00, 1.00] |
| (49,50] | 1.00 | [1.00, 1.00] | 1.00 | [1.00, 1.00] | 1.00 | [1.00, 1.00] |
| (50,51] | 1.00 | [1.00, 1.00] | 1.00 | [1.00, 1.00] | 1.00 | [1.00, 1.00] |
| (51,52] | 1.00 | [1.00, 1.00] | 1.00 | [1.00, 1.00] | 1.00 | [1.00, 1.00] |
| (52,53] | 1.00 | [1.00, 1.00] | 1.00 | [1.00, 1.00] | 1.00 | [1.00, 1.00] |
| (53,54] | 1.00 | [1.00, 1.00] | 1.00 | [1.00, 1.00] | 1.00 | [1.00, 1.00] |
| (54,55] | 1.00 | [1.00, 1.00] | 1.00 | [1.00, 1.00] | 1.00 | [1.00, 1.00] |
| (55,56] | 1.00 | [1.00, 1.00] | 1.00 | [1.00, 1.00] | 1.00 | [1.00, 1.00] |
| (56,57] | 1.00 | [1.00, 1.00] | 1.00 | [1.00, 1.00] | 1.00 | [1.00, 1.00] |
| (57,58] | 1.00 | [1.00, 1.00] | 1.00 | [1.00, 1.00] | 1.00 | [1.00, 1.00] |
| (58,59] | 1.00 | [1.00, 1.00] | 1.00 | [1.00, 1.00] | 1.00 | [1.00, 1.00] |
| (59,60] | 1.00 | [1.00, 1.00] | 1.00 | [1.00, 1.00] | 1.00 | [1.00, 1.00] |

Table S5: Standard versus high protein intake

Estimated hazard ratios (HR) and corresponding pointwise 95% confidence intervals (CI) for comparisons of protein diets by specific time intervals of the follow up (see also Figure 2 of the main document). Two different rates of protein intake were analyzed: 0.8 – 1.2 g protein/kg per day (standard protein intake) and > 1.2 g protein/kg per day (high protein intake). Three different protein diets were compared: #1: standard protein intake on days on diet #1 to #11 (early standard protein diet); #2: high protein intake on days on diet #5 to #11 (late high protein diet); #3: high protein intake on days on diet #1 to #4 (early high protein diet).

|  | Late high protein  vs. early high protein | | Early standard protein  vs. late high protein | | Early standard protein vs. early high protein | |
| --- | --- | --- | --- | --- | --- | --- |
| Interval | HR | CI | HR | CI | HR | CI |
| (4,5] | 1.00 | [1.00, 1.00] | 1.00 | [1.00, 1.00] | 1.00 | [1.00, 1.00] |
| (5,6] | 1.00 | [0.93, 1.07] | 1.00 | [1.00, 1.00] | 1.00 | [0.93, 1.07] |
| (6,7] | 1.00 | [0.88, 1.14] | 1.00 | [1.00, 1.00] | 1.00 | [0.88, 1.14] |
| (7,8] | 1.01 | [0.85, 1.19] | 1.00 | [1.00, 1.00] | 1.01 | [0.85, 1.19] |
| (8,9] | 1.02 | [0.84, 1.24] | 1.00 | [1.00, 1.00] | 1.02 | [0.84, 1.24] |
| (9,10] | 1.02 | [0.84, 1.23] | 1.02 | [0.99, 1.06] | 1.04 | [0.84, 1.28] |
| (10,11] | 1.02 | [0.84, 1.23] | 1.05 | [0.98, 1.12] | 1.07 | [0.85, 1.34] |
| (11,12] | 1.02 | [0.85, 1.22] | 1.09 | [0.99, 1.19] | 1.11 | [0.88, 1.40] |
| (12,13] | 1.02 | [0.85, 1.22] | 1.14 | [1.01, 1.28] | 1.16 | [0.91, 1.47] |
| (13,14] | 1.02 | [0.85, 1.22] | 1.20 | [1.05, 1.38] | 1.22 | [0.96, 1.55] |
| (14,15] | 1.02 | [0.90, 1.15] | 1.27 | [1.08, 1.49] | 1.29 | [1.04, 1.60] |
| (15,16] | 1.02 | [0.90, 1.15] | 1.35 | [1.12, 1.63] | 1.37 | [1.09, 1.73] |
| (16,17] | 1.02 | [0.90, 1.15] | 1.34 | [1.12, 1.61] | 1.36 | [1.09, 1.71] |
| (17,18] | 1.02 | [0.94, 1.10] | 1.33 | [1.12, 1.59] | 1.35 | [1.11, 1.66] |
| (18,19] | 1.02 | [0.94, 1.10] | 1.32 | [1.11, 1.57] | 1.34 | [1.10, 1.64] |
| (19,20] | 1.02 | [0.94, 1.10] | 1.31 | [1.11, 1.55] | 1.33 | [1.09, 1.63] |
| (20,21] | 1.01 | [0.97, 1.05] | 1.30 | [1.10, 1.54] | 1.32 | [1.10, 1.58] |
| (21,22] | 1.01 | [0.97, 1.05] | 1.29 | [1.09, 1.53] | 1.31 | [1.08, 1.57] |
| (22,23] | 1.01 | [0.97, 1.05] | 1.28 | [1.08, 1.52] | 1.30 | [1.07, 1.57] |
| (23,24] | 1.00 | [1.00, 1.00] | 1.27 | [1.07, 1.52] | 1.27 | [1.07, 1.52] |
| (24,25] | 1.00 | [1.00, 1.00] | 1.26 | [1.05, 1.52] | 1.26 | [1.05, 1.52] |
| (25,26] | 1.00 | [1.00, 1.00] | 1.25 | [1.04, 1.51] | 1.25 | [1.04, 1.51] |
| (26,27] | 1.00 | [1.00, 1.00] | 1.22 | [1.03, 1.46] | 1.22 | [1.03, 1.46] |
| (27,28] | 1.00 | [1.00, 1.00] | 1.21 | [1.01, 1.46] | 1.21 | [1.01, 1.46] |
| (28,29] | 1.00 | [1.00, 1.00] | 1.20 | [1.00, 1.46] | 1.20 | [1.00, 1.46] |
| (29,30] | 1.00 | [1.00, 1.00] | 1.17 | [0.99, 1.39] | 1.17 | [0.99, 1.39] |
| (30,31] | 1.00 | [1.00, 1.00] | 1.16 | [0.97, 1.39] | 1.16 | [0.97, 1.39] |
| (31,32] | 1.00 | [1.00, 1.00] | 1.16 | [0.96, 1.39] | 1.16 | [0.96, 1.39] |
| (32,33] | 1.00 | [1.00, 1.00] | 1.12 | [0.96, 1.32] | 1.12 | [0.96, 1.32] |
| (33,34] | 1.00 | [1.00, 1.00] | 1.12 | [0.94, 1.32] | 1.12 | [0.94, 1.32] |
| (34,35] | 1.00 | [1.00, 1.00] | 1.11 | [0.93, 1.32] | 1.11 | [0.93, 1.32] |
| (35,36] | 1.00 | [1.00, 1.00] | 1.08 | [0.93, 1.25] | 1.08 | [0.93, 1.25] |
| (36,37] | 1.00 | [1.00, 1.00] | 1.08 | [0.93, 1.25] | 1.08 | [0.93, 1.25] |
| (37,38] | 1.00 | [1.00, 1.00] | 1.07 | [0.92, 1.25] | 1.07 | [0.92, 1.25] |
| (38,39] | 1.00 | [1.00, 1.00] | 1.04 | [0.93, 1.17] | 1.04 | [0.93, 1.17] |
| (39,40] | 1.00 | [1.00, 1.00] | 1.04 | [0.92, 1.17] | 1.04 | [0.92, 1.17] |
| (40,41] | 1.00 | [1.00, 1.00] | 1.04 | [0.92, 1.17] | 1.04 | [0.92, 1.17] |
| (41,42] | 1.00 | [1.00, 1.00] | 1.02 | [0.95, 1.09] | 1.02 | [0.95, 1.09] |
| (42,43] | 1.00 | [1.00, 1.00] | 1.02 | [0.95, 1.09] | 1.02 | [0.95, 1.09] |
| (43,44] | 1.00 | [1.00, 1.00] | 1.01 | [0.94, 1.09] | 1.01 | [0.94, 1.09] |
| (44,45] | 1.00 | [1.00, 1.00] | 1.00 | [1.00, 1.00] | 1.00 | [1.00, 1.00] |
| (45,46] | 1.00 | [1.00, 1.00] | 1.00 | [1.00, 1.00] | 1.00 | [1.00, 1.00] |
| (46,47] | 1.00 | [1.00, 1.00] | 1.00 | [1.00, 1.00] | 1.00 | [1.00, 1.00] |
| (47,48] | 1.00 | [1.00, 1.00] | 1.00 | [1.00, 1.00] | 1.00 | [1.00, 1.00] |
| (48,49] | 1.00 | [1.00, 1.00] | 1.00 | [1.00, 1.00] | 1.00 | [1.00, 1.00] |
| (49,50] | 1.00 | [1.00, 1.00] | 1.00 | [1.00, 1.00] | 1.00 | [1.00, 1.00] |
| (50,51] | 1.00 | [1.00, 1.00] | 1.00 | [1.00, 1.00] | 1.00 | [1.00, 1.00] |
| (51,52] | 1.00 | [1.00, 1.00] | 1.00 | [1.00, 1.00] | 1.00 | [1.00, 1.00] |
| (52,53] | 1.00 | [1.00, 1.00] | 1.00 | [1.00, 1.00] | 1.00 | [1.00, 1.00] |
| (53,54] | 1.00 | [1.00, 1.00] | 1.00 | [1.00, 1.00] | 1.00 | [1.00, 1.00] |
| (54,55] | 1.00 | [1.00, 1.00] | 1.00 | [1.00, 1.00] | 1.00 | [1.00, 1.00] |
| (55,56] | 1.00 | [1.00, 1.00] | 1.00 | [1.00, 1.00] | 1.00 | [1.00, 1.00] |
| (56,57] | 1.00 | [1.00, 1.00] | 1.00 | [1.00, 1.00] | 1.00 | [1.00, 1.00] |
| (57,58] | 1.00 | [1.00, 1.00] | 1.00 | [1.00, 1.00] | 1.00 | [1.00, 1.00] |
| (58,59] | 1.00 | [1.00, 1.00] | 1.00 | [1.00, 1.00] | 1.00 | [1.00, 1.00] |
| (59,60] | 1.00 | [1.00, 1.00] | 1.00 | [1.00, 1.00] | 1.00 | [1.00, 1.00] |

2.5. Association of protein intake with the hazard of live hospital discharge (cause-specific hazards model)

Table S6: Low versus standard protein intake

Estimated hazard ratios (HR) and corresponding pointwise 95% confidence intervals (CI) for comparisons of protein diets by specific time intervals of the follow up (see also Figure 1 of the main document). Two different rates of protein intake were analyzed: < 0.8 g protein/kg per day (low protein intake) and 0.8 – 1.2 g protein/kg per day (standard protein intake). Three different protein diets were compared: #1: low protein intake on days on diet #1 to #11 (exclusively low protein diet); #2: standard protein intake on days on diet #5 to #11 (late standard protein diet); #3: standard protein intake on days on diet #1 to #4 (early standard protein diet).

|  | Exclusively low protein vs. early standard protein | | Exclusively low protein vs. late standard protein | | Late standard protein vs. early standard protein | |
| --- | --- | --- | --- | --- | --- | --- |
| Interval | HR | CI | HR | CI | HR | CI |
| (4,5] | 1.00 | [1.00, 1.00] | 1.00 | [1.00, 1.00] | 1.00 | [1.00, 1.00] |
| (5,6] | 0.94 | [0.79, 1.12] | 1.00 | [1.00, 1.00] | 0.94 | [0.79, 1.12] |
| (6,7] | 0.88 | [0.71, 1.08] | 1.00 | [1.00, 1.00] | 0.88 | [0.71, 1.08] |
| (7,8] | 0.86 | [0.70, 1.05] | 1.00 | [1.00, 1.00] | 0.86 | [0.70, 1.05] |
| (8,9] | 0.89 | [0.74, 1.06] | 1.00 | [1.00, 1.00] | 0.89 | [0.74, 1.06] |
| (9,10] | 0.98 | [0.83, 1.16] | 1.14 | [1.07, 1.20] | 0.87 | [0.74, 1.01] |
| (10,11] | 1.13 | [0.96, 1.33] | 1.32 | [1.20, 1.45] | 0.86 | [0.75, 1.00] |
| (11,12] | 1.32 | [1.11, 1.56] | 1.51 | [1.35, 1.70] | 0.87 | [0.75, 1.01] |
| (12,13] | 1.52 | [1.27, 1.81] | 1.70 | [1.50, 1.92] | 0.89 | [0.77, 1.04] |
| (13,14] | 1.73 | [1.43, 2.09] | 1.85 | [1.63, 2.10] | 0.93 | [0.79, 1.10] |
| (14,15] | 1.63 | [1.40, 1.89] | 1.96 | [1.72, 2.22] | 0.83 | [0.74, 0.93] |
| (15,16] | 1.72 | [1.46, 2.02] | 1.98 | [1.72, 2.28] | 0.87 | [0.77, 0.98] |
| (16,17] | 1.64 | [1.40, 1.92] | 1.79 | [1.58, 2.03] | 0.92 | [0.80, 1.05] |
| (17,18] | 1.49 | [1.30, 1.70] | 1.64 | [1.45, 1.86] | 0.91 | [0.83, 0.99] |
| (18,19] | 1.45 | [1.26, 1.66] | 1.53 | [1.35, 1.73] | 0.95 | [0.86, 1.04] |
| (19,20] | 1.43 | [1.23, 1.67] | 1.45 | [1.28, 1.64] | 0.99 | [0.88, 1.11] |
| (20,21] | 1.36 | [1.19, 1.55] | 1.38 | [1.22, 1.57] | 0.98 | [0.93, 1.04] |
| (21,22] | 1.35 | [1.18, 1.55] | 1.34 | [1.18, 1.52] | 1.01 | [0.95, 1.07] |
| (22,23] | 1.36 | [1.17, 1.58] | 1.32 | [1.16, 1.49] | 1.03 | [0.96, 1.12] |
| (23,24] | 1.31 | [1.15, 1.49] | 1.31 | [1.15, 1.49] | 1.00 | [1.00, 1.00] |
| (24,25] | 1.31 | [1.14, 1.50] | 1.31 | [1.14, 1.50] | 1.00 | [1.00, 1.00] |
| (25,26] | 1.32 | [1.14, 1.53] | 1.32 | [1.14, 1.53] | 1.00 | [1.00, 1.00] |
| (26,27] | 1.28 | [1.13, 1.47] | 1.28 | [1.13, 1.47] | 1.00 | [1.00, 1.00] |
| (27,28] | 1.29 | [1.12, 1.48] | 1.29 | [1.12, 1.48] | 1.00 | [1.00, 1.00] |
| (28,29] | 1.30 | [1.12, 1.51] | 1.30 | [1.12, 1.51] | 1.00 | [1.00, 1.00] |
| (29,30] | 1.30 | [1.13, 1.49] | 1.30 | [1.13, 1.49] | 1.00 | [1.00, 1.00] |
| (30,31] | 1.30 | [1.13, 1.50] | 1.30 | [1.13, 1.50] | 1.00 | [1.00, 1.00] |
| (31,32] | 1.31 | [1.12, 1.53] | 1.31 | [1.12, 1.53] | 1.00 | [1.00, 1.00] |
| (32,33] | 1.32 | [1.15, 1.52] | 1.32 | [1.15, 1.52] | 1.00 | [1.00, 1.00] |
| (33,34] | 1.32 | [1.14, 1.53] | 1.32 | [1.14, 1.53] | 1.00 | [1.00, 1.00] |
| (34,35] | 1.33 | [1.13, 1.57] | 1.33 | [1.13, 1.57] | 1.00 | [1.00, 1.00] |
| (35,36] | 1.32 | [1.15, 1.51] | 1.32 | [1.15, 1.51] | 1.00 | [1.00, 1.00] |
| (36,37] | 1.32 | [1.13, 1.53] | 1.32 | [1.13, 1.53] | 1.00 | [1.00, 1.00] |
| (37,38] | 1.32 | [1.11, 1.57] | 1.32 | [1.11, 1.57] | 1.00 | [1.00, 1.00] |
| (38,39] | 1.26 | [1.10, 1.44] | 1.26 | [1.10, 1.44] | 1.00 | [1.00, 1.00] |
| (39,40] | 1.26 | [1.08, 1.46] | 1.26 | [1.08, 1.46] | 1.00 | [1.00, 1.00] |
| (40,41] | 1.25 | [1.05, 1.49] | 1.25 | [1.05, 1.49] | 1.00 | [1.00, 1.00] |
| (41,42] | 1.14 | [1.01, 1.29] | 1.14 | [1.01, 1.29] | 1.00 | [1.00, 1.00] |
| (42,43] | 1.14 | [1.00, 1.31] | 1.14 | [1.00, 1.31] | 1.00 | [1.00, 1.00] |
| (43,44] | 1.14 | [0.98, 1.33] | 1.14 | [0.98, 1.33] | 1.00 | [1.00, 1.00] |
| (44,45] | 1.00 | [1.00, 1.00] | 1.00 | [1.00, 1.00] | 1.00 | [1.00, 1.00] |
| (45,46] | 1.00 | [1.00, 1.00] | 1.00 | [1.00, 1.00] | 1.00 | [1.00, 1.00] |
| (46,47] | 1.00 | [1.00, 1.00] | 1.00 | [1.00, 1.00] | 1.00 | [1.00, 1.00] |
| (47,48] | 1.00 | [1.00, 1.00] | 1.00 | [1.00, 1.00] | 1.00 | [1.00, 1.00] |
| (48,49] | 1.00 | [1.00, 1.00] | 1.00 | [1.00, 1.00] | 1.00 | [1.00, 1.00] |
| (49,50] | 1.00 | [1.00, 1.00] | 1.00 | [1.00, 1.00] | 1.00 | [1.00, 1.00] |
| (50,51] | 1.00 | [1.00, 1.00] | 1.00 | [1.00, 1.00] | 1.00 | [1.00, 1.00] |
| (51,52] | 1.00 | [1.00, 1.00] | 1.00 | [1.00, 1.00] | 1.00 | [1.00, 1.00] |
| (52,53] | 1.00 | [1.00, 1.00] | 1.00 | [1.00, 1.00] | 1.00 | [1.00, 1.00] |
| (53,54] | 1.00 | [1.00, 1.00] | 1.00 | [1.00, 1.00] | 1.00 | [1.00, 1.00] |
| (54,55] | 1.00 | [1.00, 1.00] | 1.00 | [1.00, 1.00] | 1.00 | [1.00, 1.00] |
| (55,56] | 1.00 | [1.00, 1.00] | 1.00 | [1.00, 1.00] | 1.00 | [1.00, 1.00] |
| (56,57] | 1.00 | [1.00, 1.00] | 1.00 | [1.00, 1.00] | 1.00 | [1.00, 1.00] |
| (57,58] | 1.00 | [1.00, 1.00] | 1.00 | [1.00, 1.00] | 1.00 | [1.00, 1.00] |
| (58,59] | 1.00 | [1.00, 1.00] | 1.00 | [1.00, 1.00] | 1.00 | [1.00, 1.00] |
| (59,60] | 1.00 | [1.00, 1.00] | 1.00 | [1.00, 1.00] | 1.00 | [1.00, 1.00] |

Table S7: Standard versus high protein intake

Estimated hazard ratios (HR) and corresponding pointwise 95% confidence intervals (CI) for comparisons of protein diets by specific time intervals of the follow up (see also Figure 2of the main document). Two different rates of protein intake were analyzed: 0.8 – 1.2 g protein/kg per day (standard protein intake) and > 1.2 g protein/kg per day (high protein intake). Three different protein diets were compared: #1: standard protein intake on days on diet # 1 to #11 (early standard protein diet); #2: high protein intake on days on diet #5 to #11 (late high protein diet); #3: high protein intake on days on diet #1 to #4 (early high protein diet).

|  | Late high protein  vs. early high protein | | Early standard protein  vs. late high protein | | Early standard protein  vs. early high protein | |
| --- | --- | --- | --- | --- | --- | --- |
| Interval | HR | CI | HR | CI | HR | CI |
| (4,5] | 1.00 | [1.00, 1.00] | 1.00 | [1.00, 1.00] | 1.00 | [1.00, 1.00] |
| (5,6] | 0.94 | [0.71, 1.24] | 1.00 | [1.00, 1.00] | 0.94 | [0.71, 1.24] |
| (6,7] | 0.95 | [0.68, 1.33] | 1.00 | [1.00, 1.00] | 0.95 | [0.68, 1.33] |
| (7,8] | 0.97 | [0.71, 1.33] | 1.00 | [1.00, 1.00] | 0.97 | [0.71, 1.33] |
| (8,9] | 0.94 | [0.71, 1.24] | 1.00 | [1.00, 1.00] | 0.94 | [0.71, 1.24] |
| (9,10] | 1.14 | [0.90, 1.44] | 0.74 | [0.68, 0.80] | 0.84 | [0.66, 1.07] |
| (10,11] | 1.32 | [1.06, 1.65] | 0.54 | [0.46, 0.62] | 0.71 | [0.56, 0.90] |
| (11,12] | 1.47 | [1.17, 1.85] | 0.41 | [0.34, 0.48] | 0.60 | [0.46, 0.77] |
| (12,13] | 1.57 | [1.23, 2.00] | 0.33 | [0.27, 0.39] | 0.51 | [0.39, 0.67] |
| (13,14] | 1.62 | [1.24, 2.10] | 0.28 | [0.23, 0.33] | 0.45 | [0.34, 0.59] |
| (14,15] | 1.67 | [1.42, 1.96] | 0.23 | [0.19, 0.29] | 0.39 | [0.32, 0.49] |
| (15,16] | 1.64 | [1.38, 1.94] | 0.19 | [0.15, 0.24] | 0.31 | [0.24, 0.39] |
| (16,17] | 1.56 | [1.29, 1.90] | 0.25 | [0.21, 0.30] | 0.39 | [0.31, 0.49] |
| (17,18] | 1.35 | [1.20, 1.52] | 0.32 | [0.27, 0.37] | 0.43 | [0.36, 0.51] |
| (18,19] | 1.28 | [1.12, 1.47] | 0.39 | [0.33, 0.45] | 0.50 | [0.41, 0.59] |
| (19,20] | 1.20 | [1.02, 1.42] | 0.46 | [0.39, 0.54] | 0.55 | [0.45, 0.67] |
| (20,21] | 1.08 | [1.00, 1.16] | 0.52 | [0.45, 0.61] | 0.57 | [0.48, 0.66] |
| (21,22] | 1.04 | [0.95, 1.14] | 0.58 | [0.50, 0.68] | 0.61 | [0.52, 0.72] |
| (22,23] | 1.00 | [0.90, 1.11] | 0.63 | [0.55, 0.73] | 0.63 | [0.53, 0.76] |
| (23,24] | 1.00 | [1.00, 1.00] | 0.67 | [0.58, 0.78] | 0.67 | [0.58, 0.78] |
| (24,25] | 1.00 | [1.00, 1.00] | 0.69 | [0.59, 0.81] | 0.69 | [0.59, 0.81] |
| (25,26] | 1.00 | [1.00, 1.00] | 0.70 | [0.59, 0.83] | 0.70 | [0.59, 0.83] |
| (26,27] | 1.00 | [1.00, 1.00] | 0.75 | [0.65, 0.87] | 0.75 | [0.65, 0.87] |
| (27,28] | 1.00 | [1.00, 1.00] | 0.76 | [0.65, 0.89] | 0.76 | [0.65, 0.89] |
| (28,29] | 1.00 | [1.00, 1.00] | 0.77 | [0.65, 0.91] | 0.77 | [0.65, 0.91] |
| (29,30] | 1.00 | [1.00, 1.00] | 0.80 | [0.69, 0.93] | 0.80 | [0.69, 0.93] |
| (30,31] | 1.00 | [1.00, 1.00] | 0.80 | [0.68, 0.94] | 0.80 | [0.68, 0.94] |
| (31,32] | 1.00 | [1.00, 1.00] | 0.80 | [0.67, 0.95] | 0.80 | [0.67, 0.95] |
| (32,33] | 1.00 | [1.00, 1.00] | 0.80 | [0.69, 0.93] | 0.80 | [0.69, 0.93] |
| (33,34] | 1.00 | [1.00, 1.00] | 0.80 | [0.68, 0.94] | 0.80 | [0.68, 0.94] |
| (34,35] | 1.00 | [1.00, 1.00] | 0.80 | [0.66, 0.96] | 0.80 | [0.66, 0.96] |
| (35,36] | 1.00 | [1.00, 1.00] | 0.79 | [0.68, 0.92] | 0.79 | [0.68, 0.92] |
| (36,37] | 1.00 | [1.00, 1.00] | 0.79 | [0.66, 0.93] | 0.79 | [0.66, 0.93] |
| (37,38] | 1.00 | [1.00, 1.00] | 0.78 | [0.64, 0.95] | 0.78 | [0.64, 0.95] |
| (38,.39] | 1.00 | [1.00, 1.00] | 0.80 | [0.68, 0.94] | 0.80 | [0.68, 0.94] |
| (39,40] | 1.00 | [1.00, 1.00] | 0.79 | [0.66, 0.95] | 0.79 | [0.66, 0.95] |
| (40,41] | 1.00 | [1.00, 1.00] | 0.78 | [0.63, 0.97] | 0.78 | [0.63, 0.97] |
| (41.42] | 1.00 | [1.00, 1.00] | 0.85 | [0.73, 1.00] | 0.85 | [0.73, 1.00] |
| (42,43] | 1.00 | [1.00, 1.00] | 0.85 | [0.71, 1.01] | 0.85 | [0.71, 1.01] |
| (43,44] | 1.00 | [1.00, 1.00] | 0.84 | [0.69, 1.03] | 0.84 | [0.69, 1.03] |
| (44,45] | 1.00 | [1.00, 1.00] | 1.00 | [1.00, 1.00] | 1.00 | [1.00, 1.00] |
| (45,46] | 1.00 | [1.00, 1.00] | 1.00 | [1.00, 1.00] | 1.00 | [1.00, 1.00] |
| (46,47] | 1.00 | [1.00, 1.00] | 1.00 | [1.00, 1.00] | 1.00 | [1.00, 1.00] |
| (47,48] | 1.00 | [1.00, 1.00] | 1.00 | [1.00, 1.00] | 1.00 | [1.00, 1.00] |
| (48,49] | 1.00 | [1.00, 1.00] | 1.00 | [1.00, 1.00] | 1.00 | [1.00, 1.00] |
| (49,50] | 1.00 | [1.00, 1.00] | 1.00 | [1.00, 1.00] | 1.00 | [1.00, 1.00] |
| (50,51] | 1.00 | [1.00, 1.00] | 1.00 | [1.00, 1.00] | 1.00 | [1.00, 1.00] |
| (51,52] | 1.00 | [1.00, 1.00] | 1.00 | [1.00, 1.00] | 1.00 | [1.00, 1.00] |
| (52,53] | 1.00 | [1.00, 1.00] | 1.00 | [1.00, 1.00] | 1.00 | [1.00, 1.00] |
| (53,54] | 1.00 | [1.00, 1.00] | 1.00 | [1.00, 1.00] | 1.00 | [1.00, 1.00] |
| (54,55] | 1.00 | [1.00, 1.00] | 1.00 | [1.00, 1.00] | 1.00 | [1.00, 1.00] |
| (55,56] | 1.00 | [1.00, 1.00] | 1.00 | [1.00, 1.00] | 1.00 | [1.00, 1.00] |
| (56,57] | 1.00 | [1.00, 1.00] | 1.00 | [1.00, 1.00] | 1.00 | [1.00, 1.00] |
| (57,58] | 1.00 | [1.00, 1.00] | 1.00 | [1.00, 1.00] | 1.00 | [1.00, 1.00] |
| (58,59] | 1.00 | [1.00, 1.00] | 1.00 | [1.00, 1.00] | 1.00 | [1.00, 1.00] |
| (59,60] | 1.00 | [1.00, 1.00] | 1.00 | [1.00, 1.00] | 1.00 | [1.00, 1.00] |

- 1. Extended primary analysis

Table S8: Definition of comparisons of different hypothetical protein diets

| Title of Comparison | Definition of Comparison |
| --- | --- |
| Comparison A | Exclusively low protein diet (intake on days on diet #5 to #11: < 0.8 g protein/kg per day, level I) vs. late standard protein diet (intake on days on diet #5 to #11: 0.8 – 1.2 g protein/kg per day, level II) |
| Comparison B | late standard protein diet (intake on days on diet #1 to #4: < 0.8 g protein/kg per day, level I) vs. early standard protein diet (intake on days on diet #1 to #4: 0.8 – 1.2 g protein/kg per day) |
| Comparison C | Exclusively low protein diet (intake on days on diet #1 to #11: < 0.8 g protein/kg per day, level I) vs. early standard protein diet (intake on days on diet #1 to #11: 0.8 – 1.2 g protein/kg per day, level II) |
| Comparison D | Early standard protein diet (intake on days on diet #5 to #11: 0.8 – 1.2 g protein/kg per day, level II) vs. late high protein diet (intake on days on diet #5 to #11: > 1.2 g protein/kg per day, level III) |
| Comparison E | late high protein diet (intake on days on diet #1 to #4: 0.8 – 1.2 g protein/kg per day, level II) vs. early high protein diet (intake on days on diet #1 to #4: > 1.2 g protein/kg per day, level III) |
| Comparison F | Early standard protein diet (intake on days on diet #1 to #11: 0.8 – 1.2 g protein/kg per day, level II) vs. early high protein diet (intake on days on diet 1 to 11: > 1.2 g protein/kg per day, level III) |

### 2.6.1. Subdistribution hazards model (dynamic time window) to estimate outcome.

Figure S5

Time-varying associations of different hypothetical protein diets with the rate of in-hospital death (dynamic time window). Instead of calculating cause-specific hazards, we now calculated subdistribution hazards. Definitions of protein intakes and diet comparisons are given in Table S8. Reference protein diet is the one which provides lesser protein (e.g., a hazard ratio < 1 (and 95% confidence interval) would indicate that the risk associated with the protein diet providing more protein was smaller). Please note that HRs (and pointwise corresponding 95% CIs) must be 1 for the first time interval (due to the specification of the model), and for time intervals, in which protein intake of both hypothetical protein diets are identical.


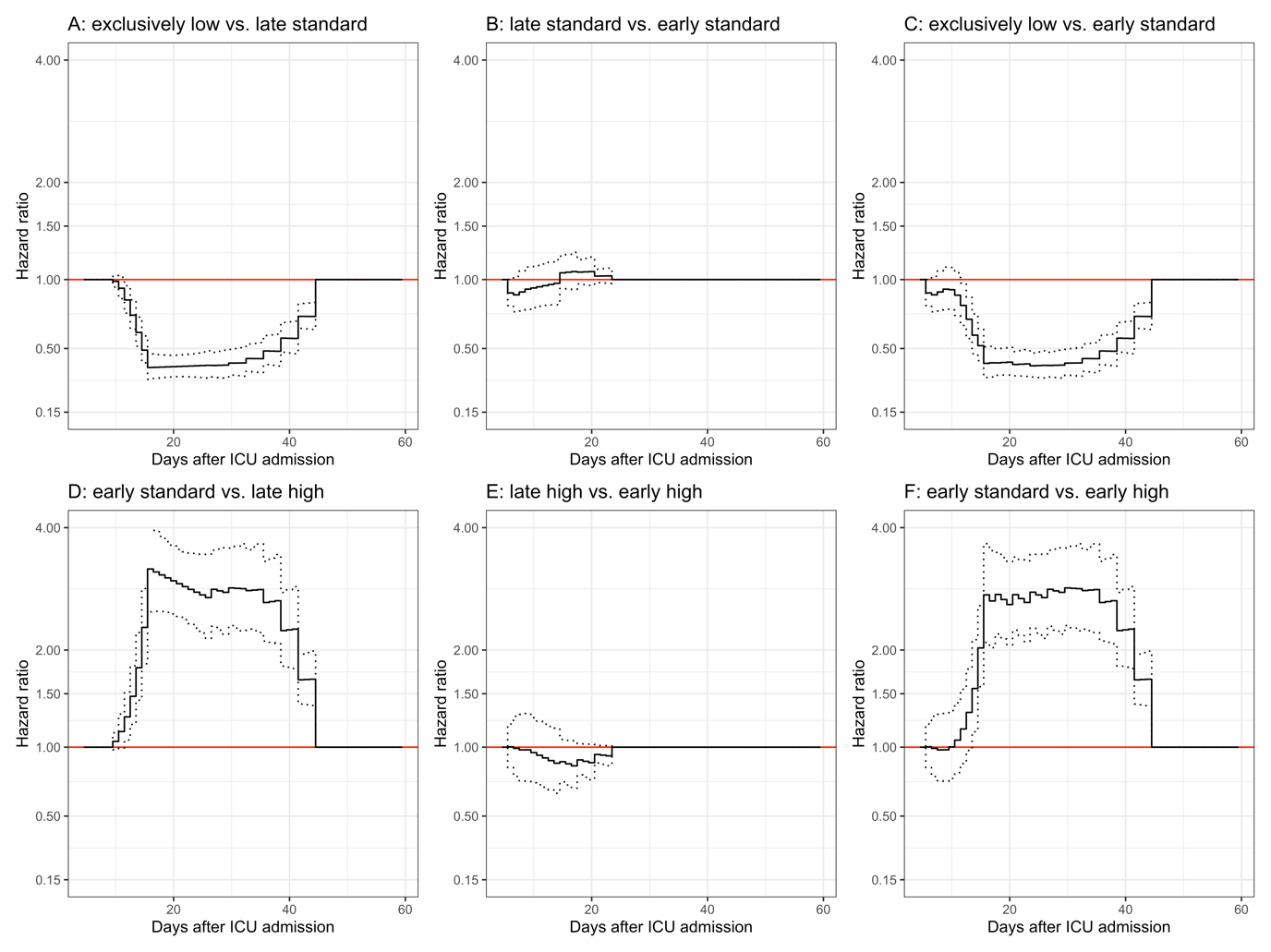


2.6.2. Cumulative incidence functions (CIF) (dynamic time window)

2.6.2.1. Cause-specific hazard (in-hospital death)

Figure S6:

Cumulative Incidence Function (CIF) with 95% confidence bands for the various hypothetical nutrition protocols. The CIF depicts the estimated proportion of patients that have experienced the event of interest (here in-hospital death) until a certain time point (days after ICU admission), given the respective diet. Note that the CIF also depends on the value of the other covariates, which were set to mean values for numeric covariates and to modus values for categorical covariates.


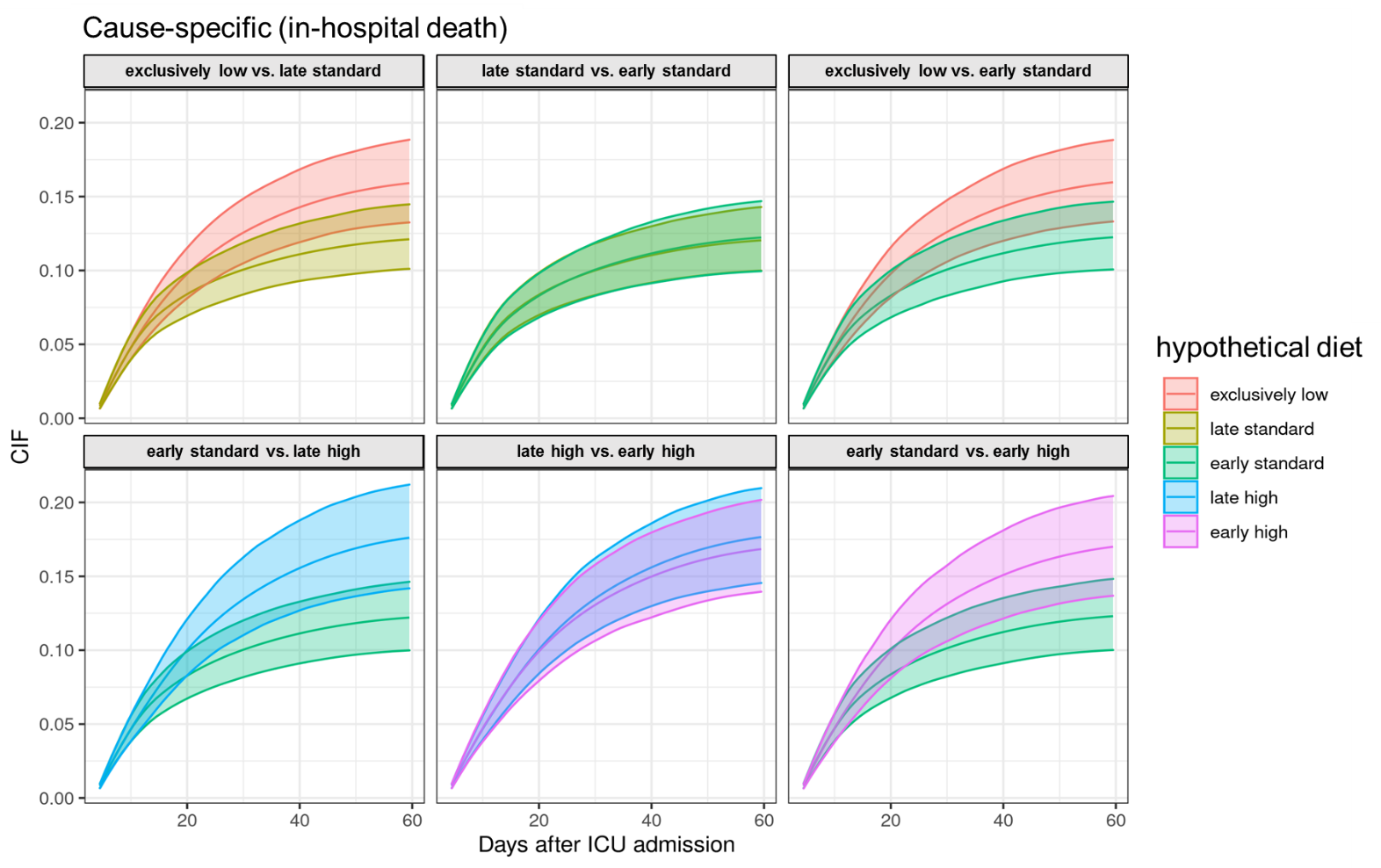


2.6.2.2. Cause-specific hazard (live hospital discharge)

Figure S7:

Cumulative Incidence Function (CIF) with 95% confidence bands for the various hypothetical protein diets. The CIF depicts the estimated proportion of patients that have experienced the event of interest (here live hospital discharge) until a certain time point (days after ICU admission), given the respective diet. Note that the CIF also depends on the value of the other covariates, which were set to mean values for numeric covariates and to modus values for categorical covariates.

###
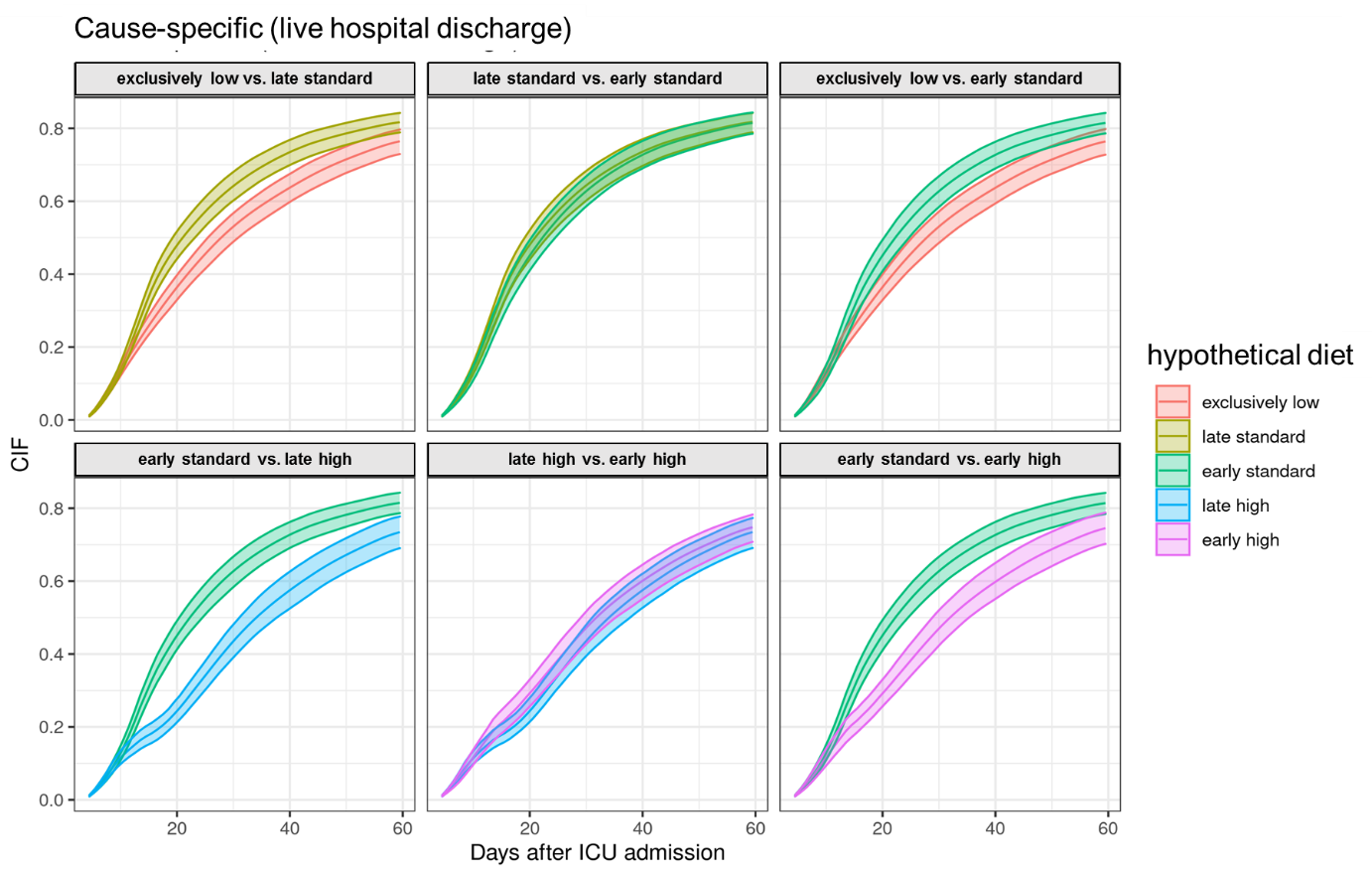


### 2.6.2.3. Subdistributional hazard (in-hospital death)

Figure S8:

Cumulative Incidence Function (CIF) with 95% confidence bands for the various hypothetical protein diets. The CIF depicts the estimated proportion of patients that have experienced the event of interest (here in-hospital death) until a certain time point (days after ICU admission), given the respective diet. Note that the CIF also depends on the value of the other covariates, which were set to mean values for numeric covariates and to modus values for categorical covariates.

**
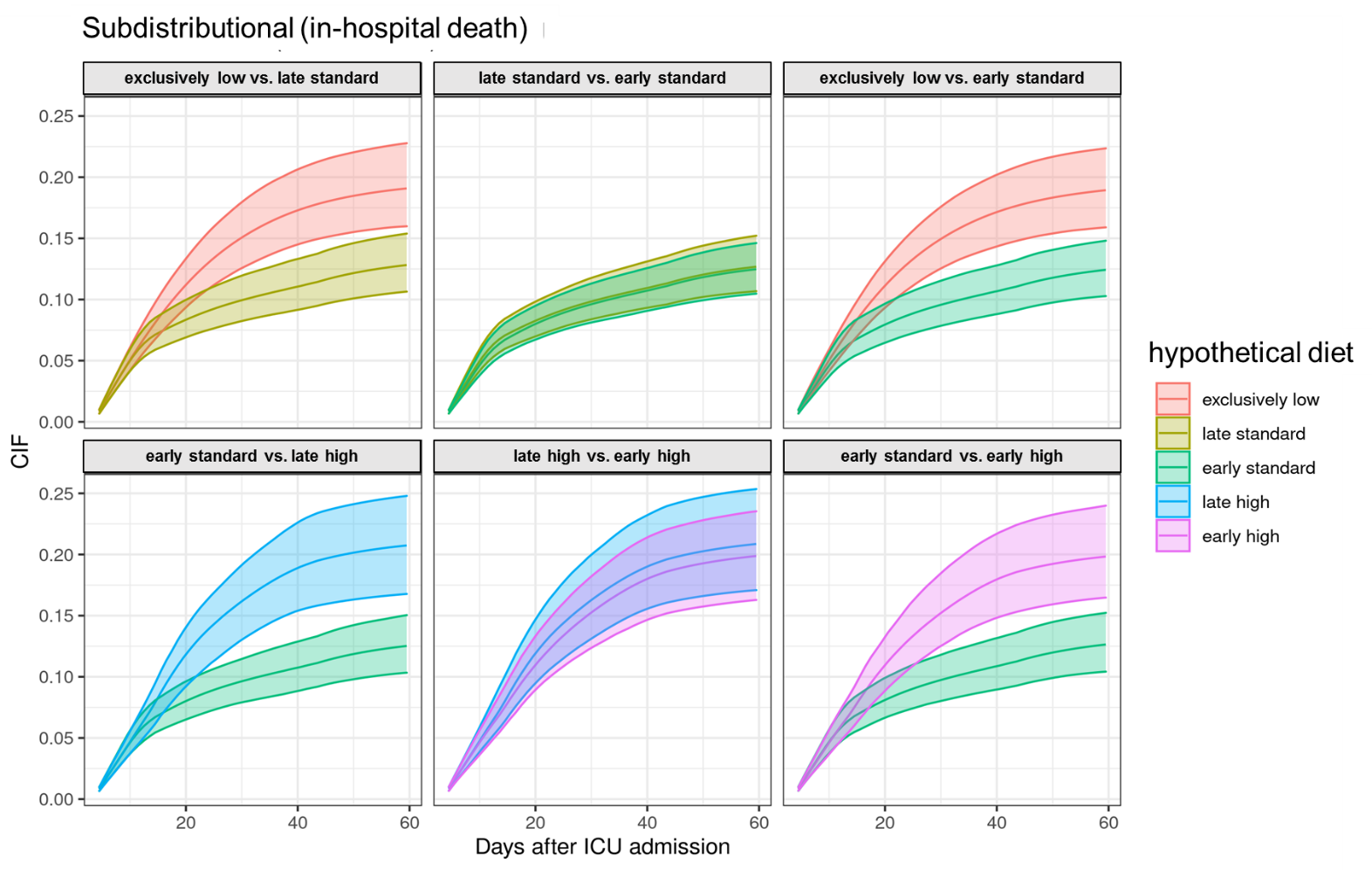
**

- 1. Sensitivity analysis

### 2.7.1. Cause-specific hazards models using a static time window to estimate outcome.

For the primary analysis we had used a dynamic time window. Figures S9 and S10 show the results of a sensitivity analysis where we used a static time window assuming that even short periods of artificial protein intake affected outcome up to day 60 after ICU admission.

Figure S9:

Time-varying associations of different hypothetical protein diets with the rate of in-hospital death (static time window). Definitions of protein intakes and diet comparisons are given in Table S8. Reference protein diet is the one which provides lesser protein (e.g., a hazard ratio < 1 (and 95% confidence interval) would indicate that the risk associated with the protein diet providing more protein was smaller). Please note that HRs (and pointwise corresponding 95% CIs) must be 1 for the first time interval (due to the specification of the model), and for time intervals, in which protein intake of both hypothetical protein diets are identical. Please also note that due to methodological restrictions, results beyond day 30 become increasingly uncertain.


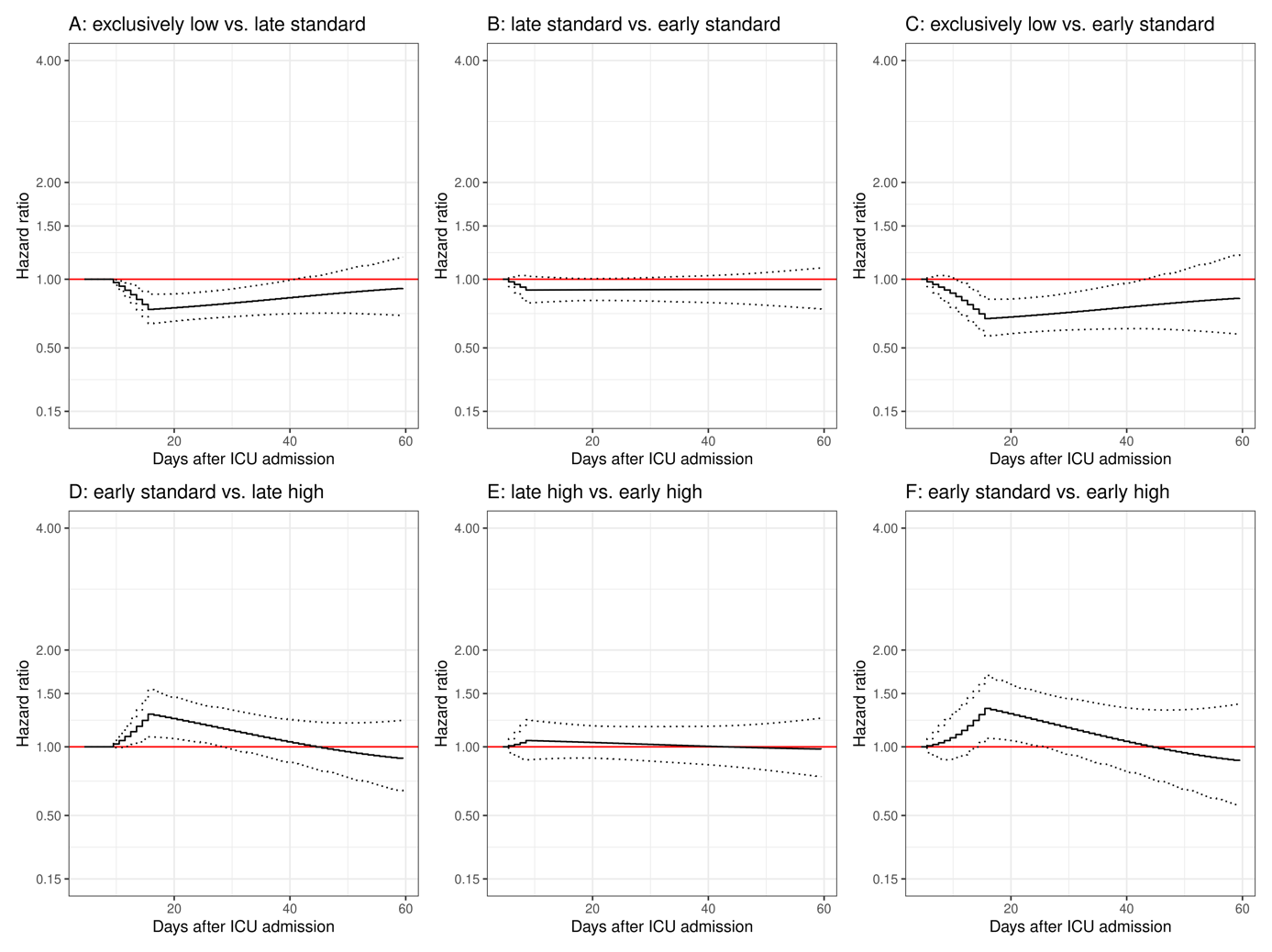


Figure S10:

Time-varying associations of different hypothetical protein diets with the rate of live hospital discharge (static time window). Definitions of protein intakes and diet comparisons are given in Table S8. Reference protein diet is the one which provides lesser protein (e.g., a hazard ratio < 1 (and 95% confidence interval) would indicate that the risk associated with the protein diet providing more protein was smaller). Please note that HRs (and pointwise corresponding 95% CIs) must be 1 for the first time interval (due to the specification of the model), and for time intervals, in which protein intake of both hypothetical protein diets are identical. Please also note that due to methodological restrictions, results beyond day 30 become increasingly uncertain.


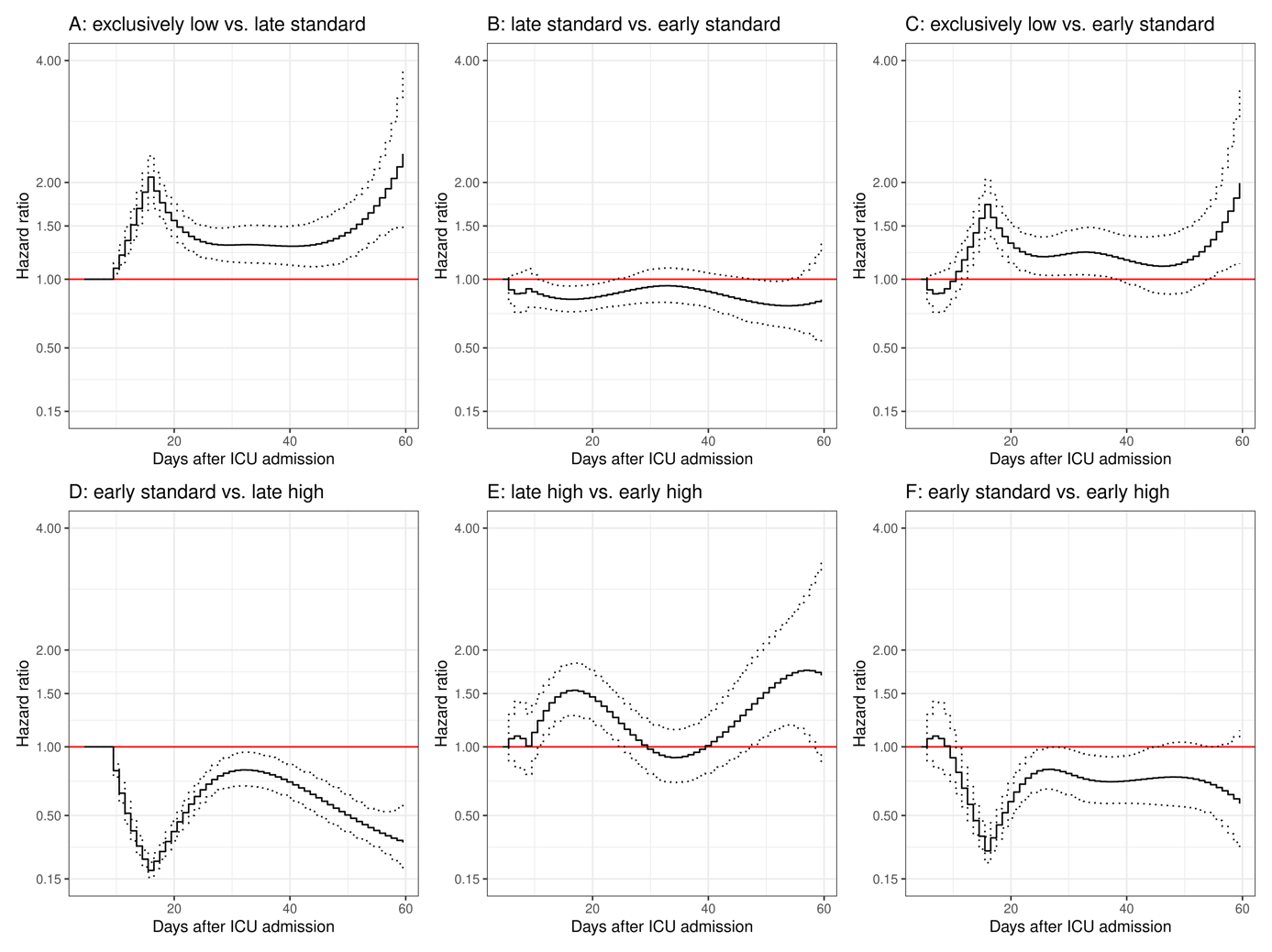


### 2.7.2. In-ICU death and live ICU discharge as competing risks

For the primary analysis we had used in-hospital death and live hospital discharge as competing risks**.** To perform this analysis, it was necessary to assume a daily standard protein intake for those patients, who had been discharged alive from the ICU before day 11 after ICU admission. By limiting our analysis to in-ICU death and live ICU discharge, we eliminated the need for an additional imputation of protein intake. Figures S11 - S14 show the results of a sensitivity analysis where we used these alternative definitions of competing risks.

2.7.2.1 Cause-specific hazards models (dynamic time window)

Figure S11: Comparison of an early or late standard protein intake with a low protein intake

Column 1: design of diet comparisons analyzing different hypothetical protein diets (Table 1). Protein intake reflects the median of corresponding categories (standard: 0.8 – 1.2 g protein/kg per day; low: < 0.8 g/kg per day).

Column 2 and 3: corresponding time-varying associations of different hypothetical diets with the rate of in-ICU death or live ICU discharge (cause-specific hazards).

Solid lines indicate hazard ratios (HR), hatched lines indicate corresponding 95% confidence intervals (CI). Reference diet is that which provides fewer protein (e.g., an HR (and 95% CI) < 1 would indicate that the rate of in-ICU death/live ICU discharge associated with the diet providing more protein was smaller). Please note that HRs (and corresponding 95% CIs) must be 1 for the first time interval between day 4 and 5 (due to the specification of the lag time), and also for time intervals, in which protein intake of both hypothetical diets is identical within the relevant time window that affects the hazard.


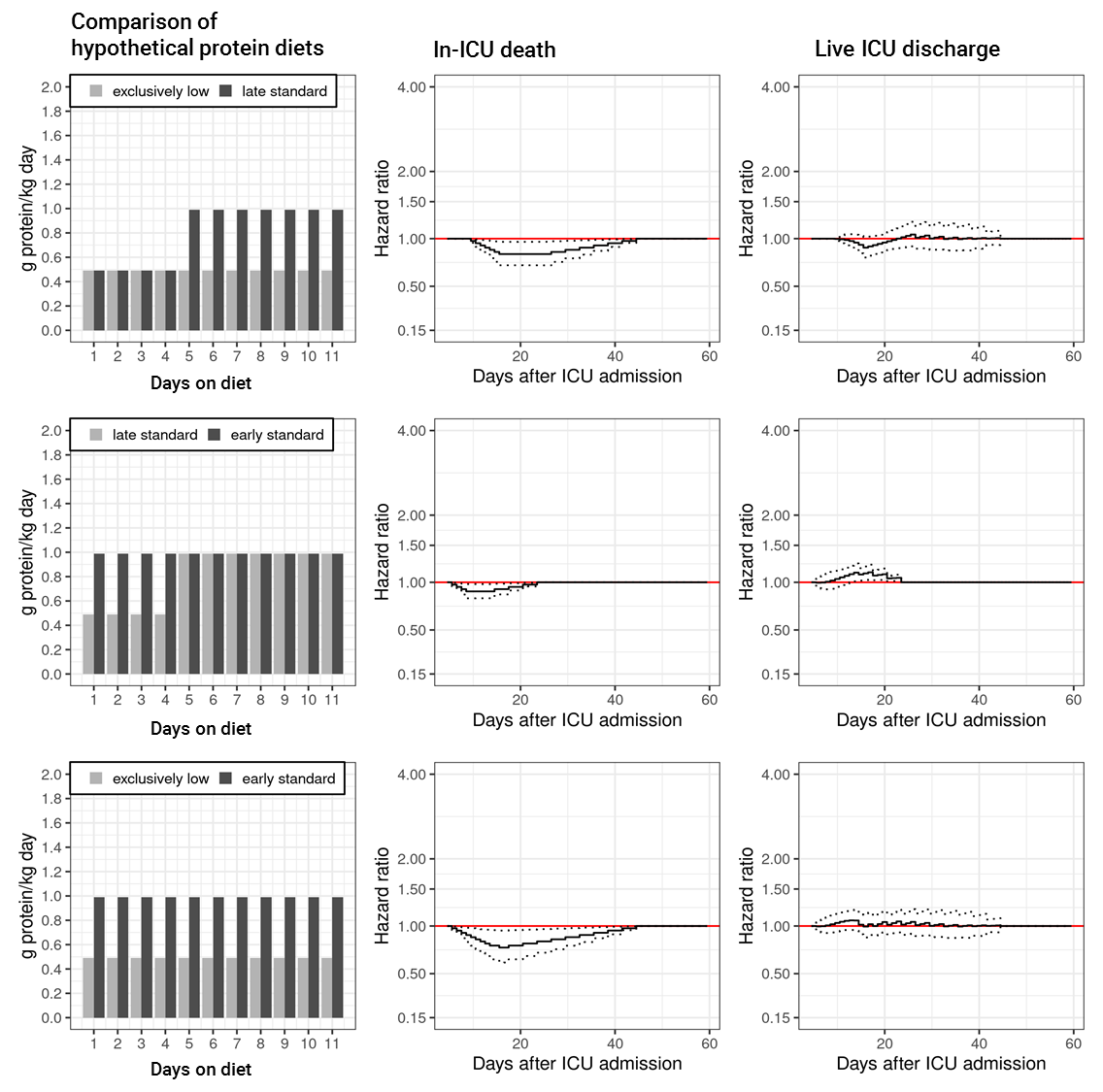


Figure S12: Comparison of an early or late high protein intake with a standard protein intake

Column 1: design of diet comparisons analyzing different hypothetical protein diets (Table 1). Protein intake reflects the median of corresponding categories (standard: 0.8 – 1.2 g protein/kg per day; high: > 1.2 g/kg per day).

Column 2 and 3: corresponding time-varying associations of different hypothetical diets with the rate of in-ICU death or live ICU discharge (cause-specific hazards).

Solid lines indicate hazard ratios (HR), hatched lines indicate corresponding 95% confidence intervals (CI). Reference diet is that which provides fewer protein (e.g., an HR (and 95% CI) < 1 would indicate that rate of in-ICU death/live ICU discharge associated with the diet providing more protein was smaller). Please note that HRs (and corresponding 95% CIs) must be 1 for the first time interval between day 4 and 5 (due to the specification of the lag time), and also for time intervals, in which protein intake of both hypothetical diets is identical within the relevant time window that affects the hazard.


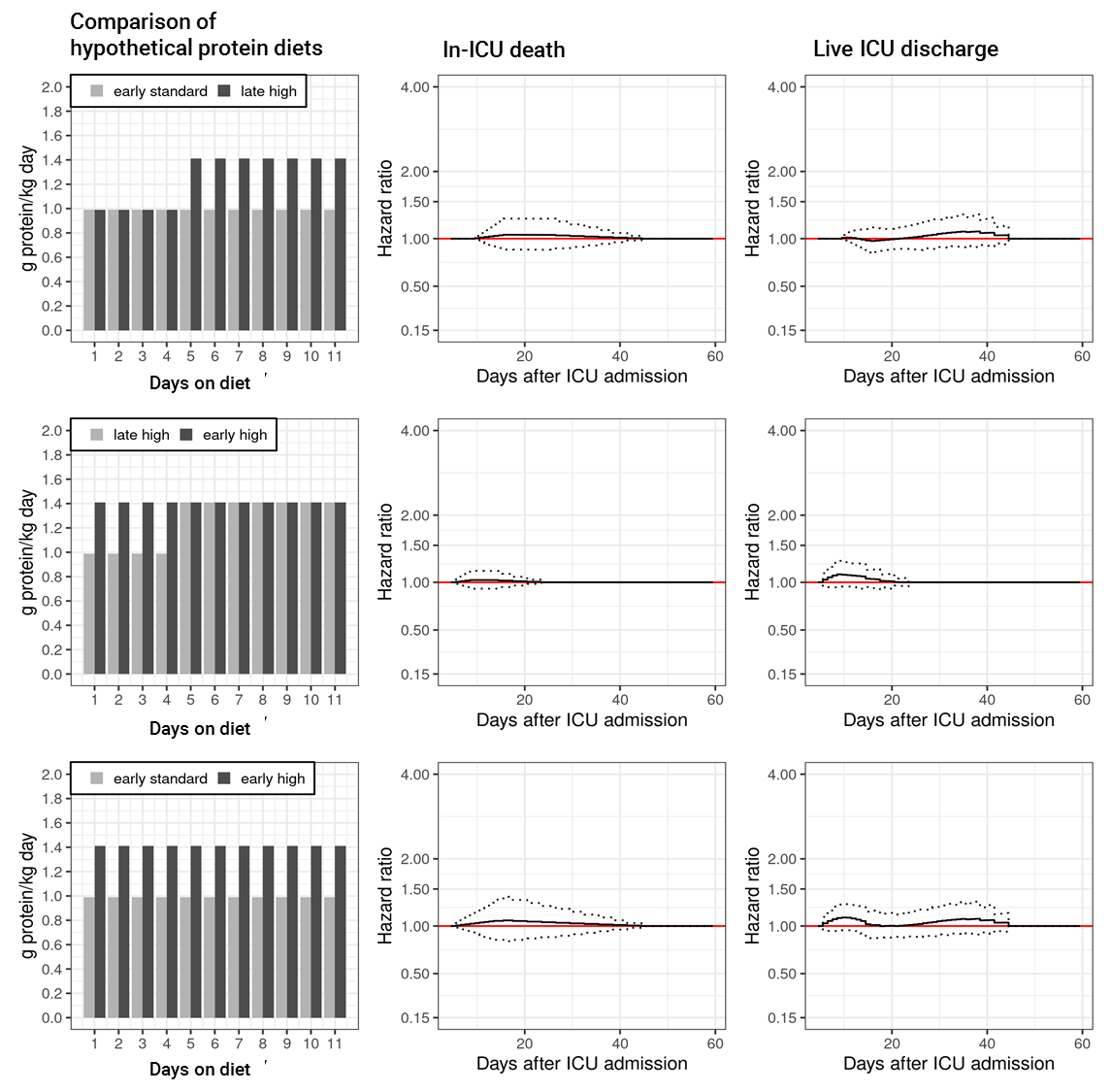


2.7.2.2 Cause-specific cumulative incidence function (dynamic time window)

Figure S13: In-ICU death

Cumulative Incidence Function (CIF) with 95% confidence bands for the various hypothetical diets. The CIF depicts the estimated proportion of patients that have experienced the event of interest (here in-ICU death) until a certain time point (days after ICU admission), given the respective diet. Note that the CIF also depends on the value of the other covariates, which were set to mean values for numeric covariates and to modus values for categorical covariates.


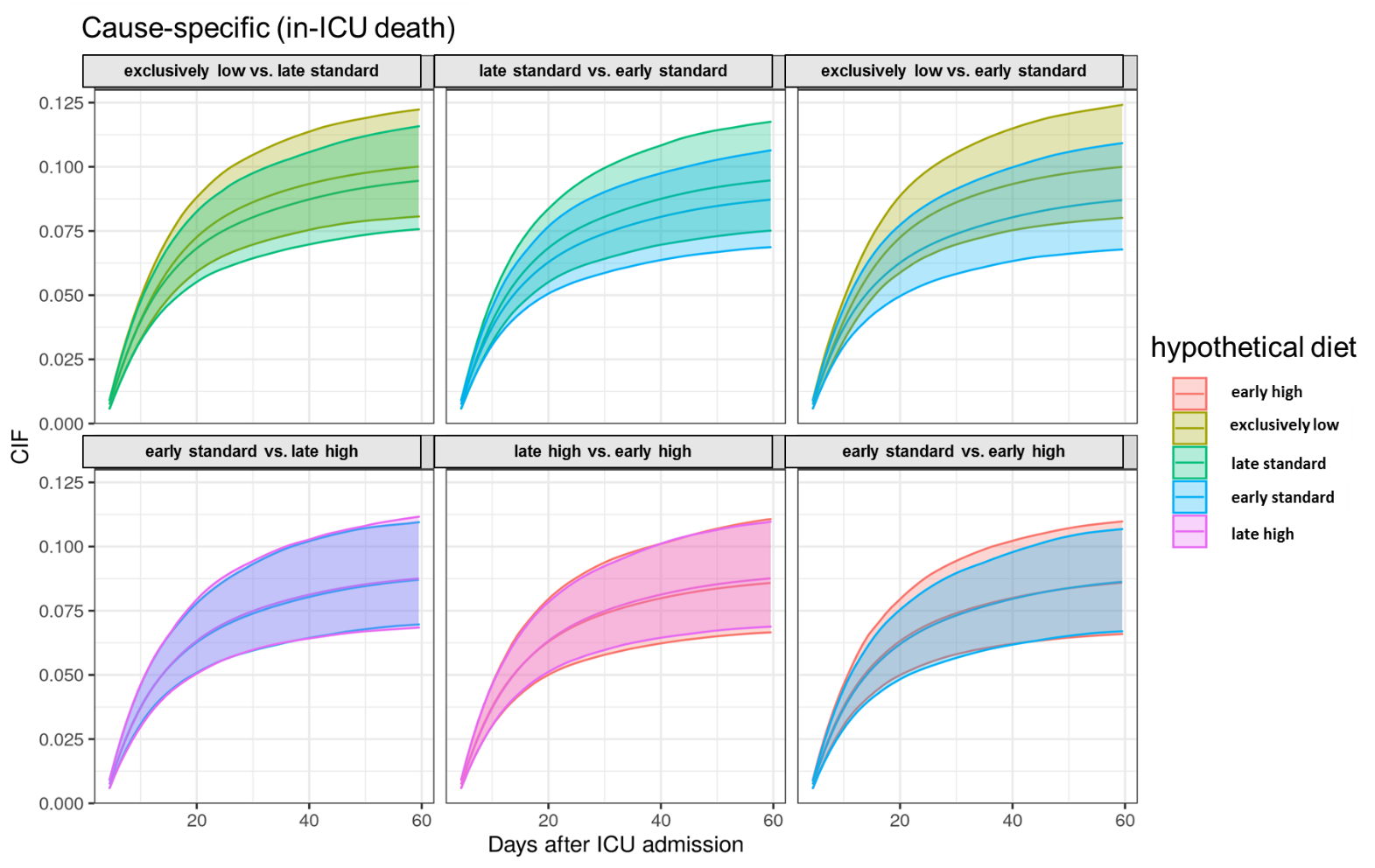


Figure S14: Live ICU discharge

Cumulative Incidence Function (CIF) with 95% confidence bands for the various hypothetical protein diets. The CIF depicts the estimated proportion of patients that have experienced the event of interest (here in-ICU death) until a certain time point (days after ICU admission), given the respective diet. Note that the CIF also depends on the value of the other covariates, which were set to mean values for numeric covariates and to modus values for categorical covariates.


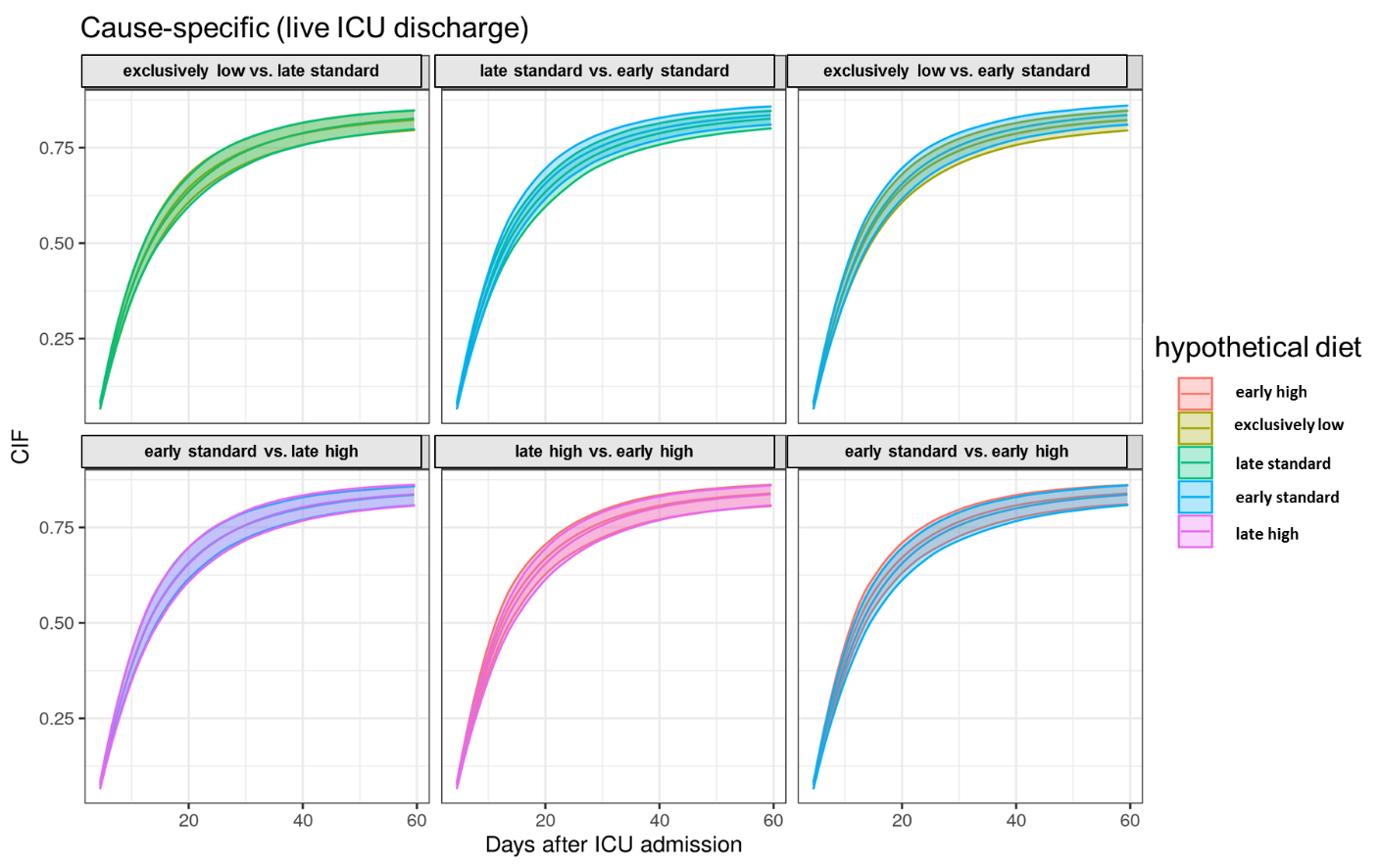


- 1. Subgroup analysis in obese patients

Figure S15:

Time-varying associations of different hypothetical protein diets with the rate of in-hospital death (dynamic time window, cause specific hazard). Definitions of protein intakes and diet comparisons are given in Table S8. Reference protein diet is the one which provides lesser protein (e.g., a hazard ratio < 1 (and 95% confidence interval) would indicate that the risk associated with the protein diet providing more protein was smaller). Please note that HRs (and pointwise corresponding 95% CIs) must be 1 for the first time interval (due to the specification of the model), and for time intervals, in which protein intake of both hypothetical protein diets are identical. Please also note that due to methodological restrictions, results beyond day 30 become increasingly uncertain.


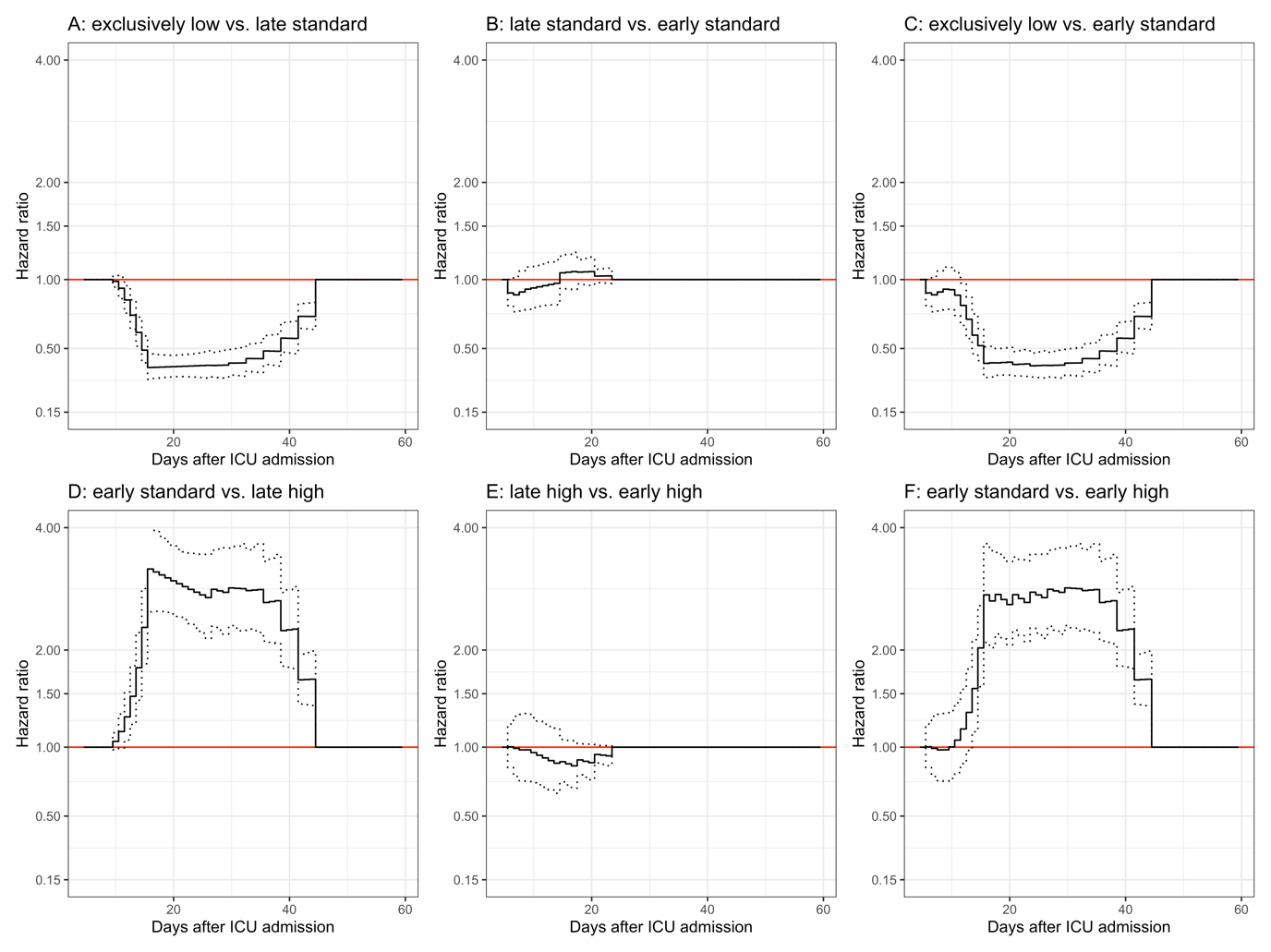


Figure S16:

Time-varying associations of different hypothetical protein diets with the rate of live hospital discharge (dynamic time window, cause specific hazard). Definitions of protein intakes and diet comparisons are given in Table S8. Reference protein diet is the one which provides lesser protein (e.g., a hazard ratio < 1 (and 95% confidence interval) would indicate that the risk associated with the protein diet providing more protein was smaller). Please note that HRs (and pointwise corresponding 95% CIs) must be 1 for the first time interval (due to the specification of the model), and for time intervals, in which protein intake of both hypothetical protein diets are identical. Please also note that due to methodological restrictions, results beyond day 30 become increasingly uncertain.


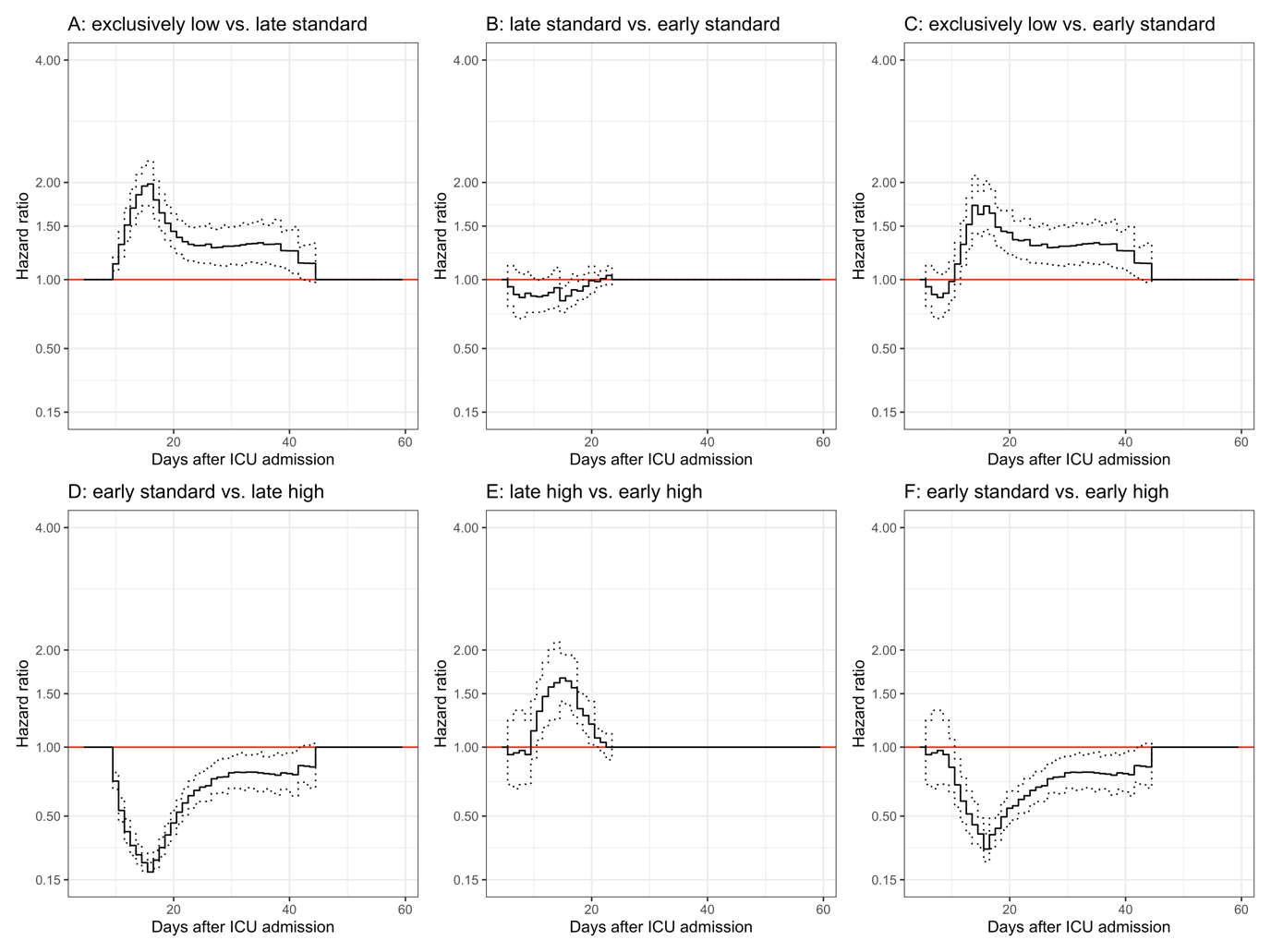


- 1. Subgroup analysis in patients with a different total calorie intake on day 3 after ICU admission

Figure S17:

Comparison of an early or late standard protein intake with a low protein intake in patients with a low calorie intake (< 30% of target) on day 3 after ICU admission

Column 1: design of diet comparisons analyzing different hypothetical protein diets (pseudo-observations) (Table 1). Protein intake reflects the median of corresponding categories (standard: 0.8 – 1.2 g protein/kg per day; low: < 0.8 g/kg per day).

Column 2 and 3: corresponding time-varying associations of different hypothetical diets with the hazard of in-hospital death or live hospital discharge (cause-specific hazards).

Solid lines indicate hazard ratios (HR), hatched lines indicate corresponding 95% confidence intervals (CI) (HRs and CIs for specific time intervals after ICU admission are presented in Tables S4 and S6 of the Additional File). Reference diet is that which provides fewer protein (e.g., an HR (and 95% CI) < 1 would indicate that the hazard of in-hospital death/live hospital discharge associated with the diet providing more protein was smaller). Please note that HRs (and corresponding 95% CIs) must be 1 for the first time interval between day 4 and 5 (due to the specification of the lag time), and also for time intervals, in which protein intake of both hypothetical diets is identical within the relevant time window that affects the hazard.


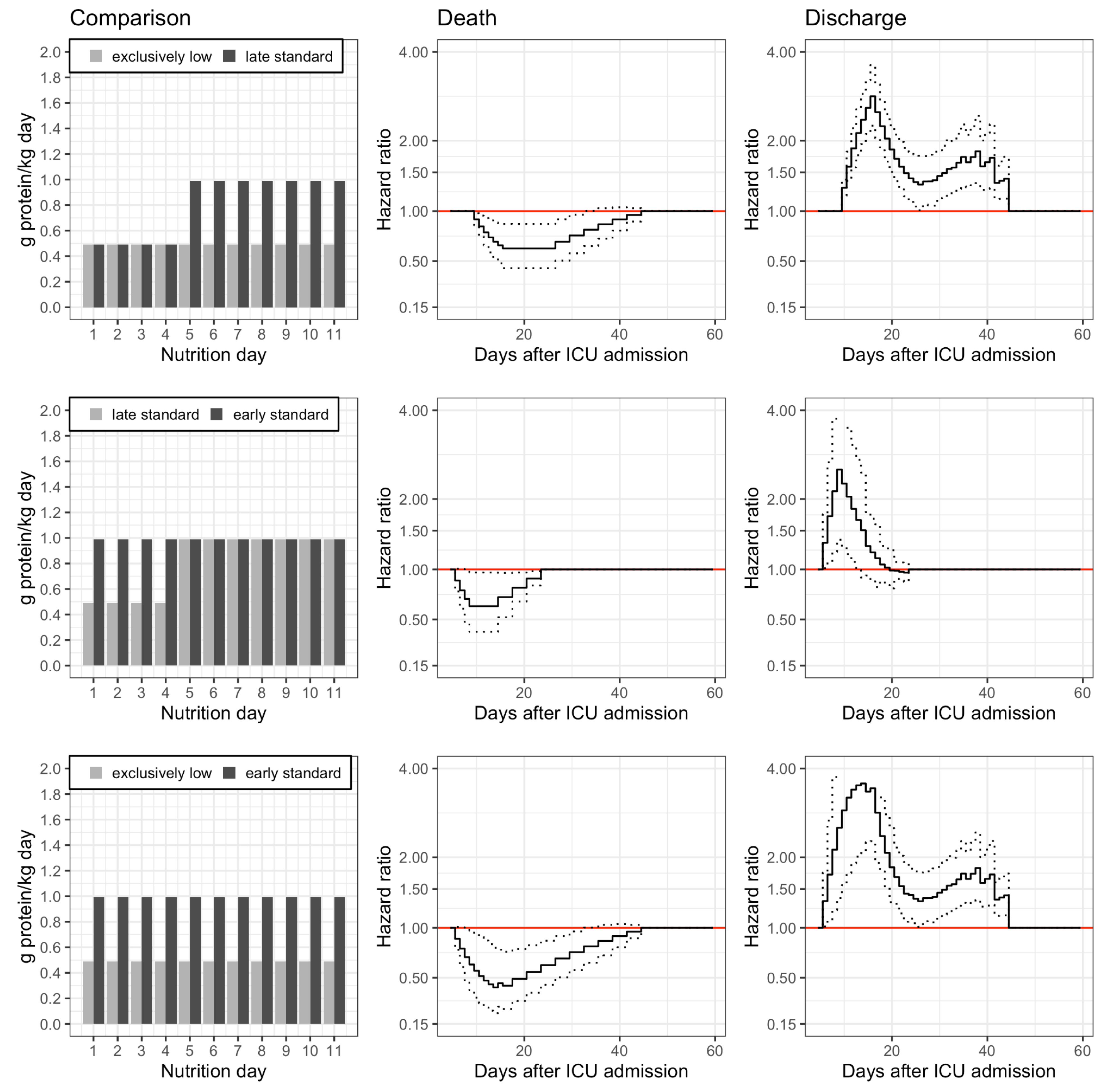


Figure S18:

Comparison of an early or late high protein intake with a standard protein intake in patients with a low calorie intake (< 30% of target) on day 3 after ICU admission

Column 1: design of diet comparisons analyzing different hypothetical protein diets (pseudo-observations) (Table 1). Protein intake reflects the median of corresponding categories (standard: 0.8 – 1.2 g protein/kg per day; high: > 1.2 g/kg per day).

Column 2 and 3: corresponding time-varying associations of different hypothetical diets with the rate of in-hospital death or live hospital discharge (cause-specific hazards).

Solid lines indicate hazard ratios (HR), hatched lines indicate corresponding 95% confidence intervals (CI) (HRs and CIs for specific time intervals after ICU admission are presented in S6 and S7 of the Additional File). Reference diet is that which provides fewer protein (e.g., an HR (and 95% CI) < 1 would indicate that rate of in-hospital death/live hospital discharge associated with the diet providing more protein was smaller). Please note that HRs (and corresponding 95% CIs) must be 1 for the first time interval between day 4 and 5 (due to the specification of the lag time), and also for time intervals, in which protein intake of both hypothetical diets is identical within the relevant time window that affects the hazard.


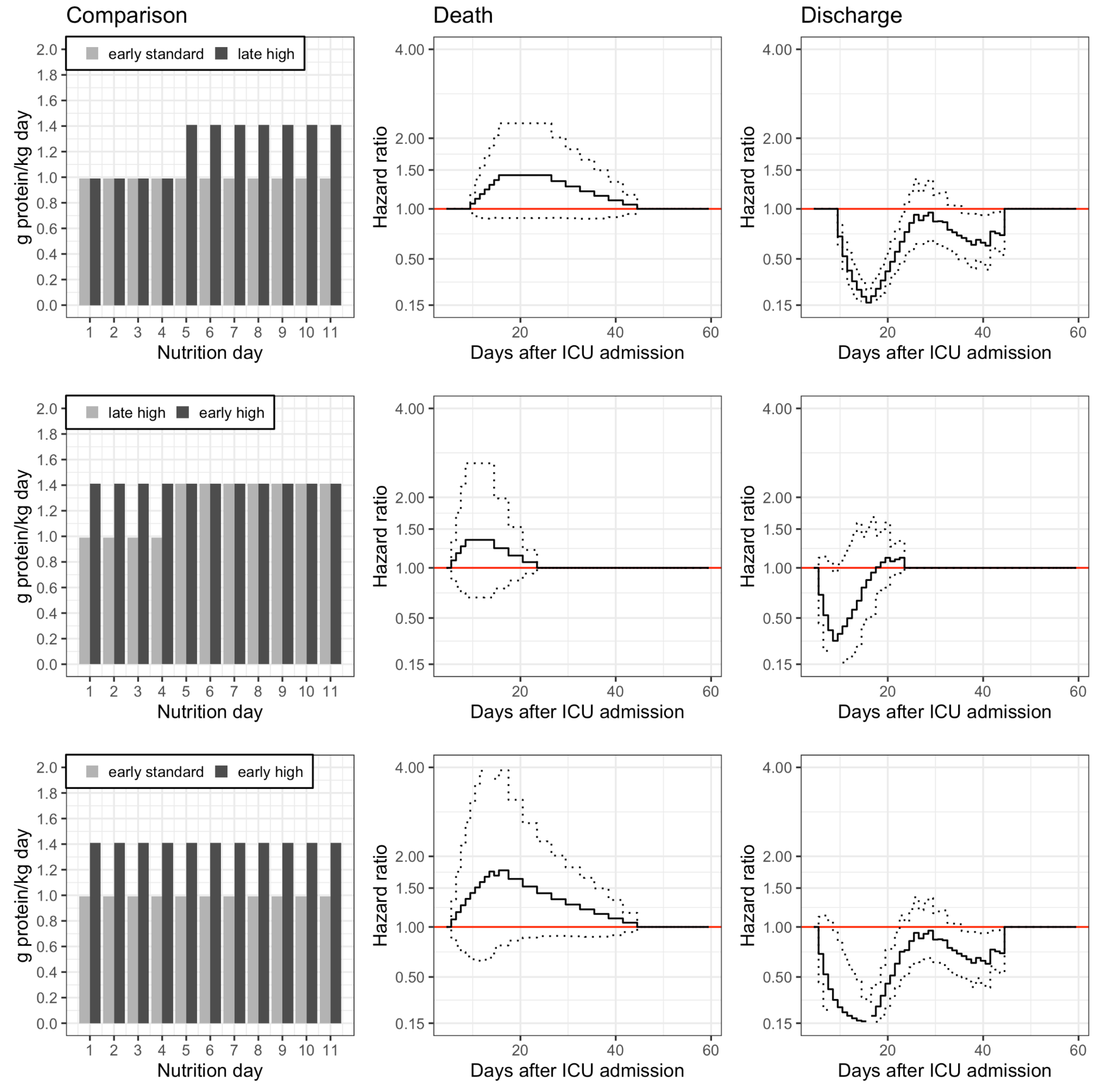


Figure S19:

Comparison of an early or late standard protein intake with a low protein intake in patients with a moderate calorie intake (30 – 70 % of target) on day 3 after ICU admission

Column 1: design of diet comparisons analyzing different hypothetical protein diets (pseudo-observations) (Table 1). Protein intake reflects the median of corresponding categories (standard: 0.8 – 1.2 g protein/kg per day; low: < 0.8 g/kg per day).

Column 2 and 3: corresponding time-varying associations of different hypothetical diets with the hazard of in-hospital death or live hospital discharge (cause-specific hazards).

Solid lines indicate hazard ratios (HR), hatched lines indicate corresponding 95% confidence intervals (CI) (HRs and CIs for specific time intervals after ICU admission are presented in Tables S4 and S6 of the Additional File). Reference diet is that which provides fewer protein (e.g., an HR (and 95% CI) < 1 would indicate that the hazard of in-hospital death/live hospital discharge associated with the diet providing more protein was smaller). Please note that HRs (and corresponding 95% CIs) must be 1 for the first time interval between day 4 and 5 (due to the specification of the lag time), and also for time intervals, in which protein intake of both hypothetical diets is identical within the relevant time window that affects the hazard


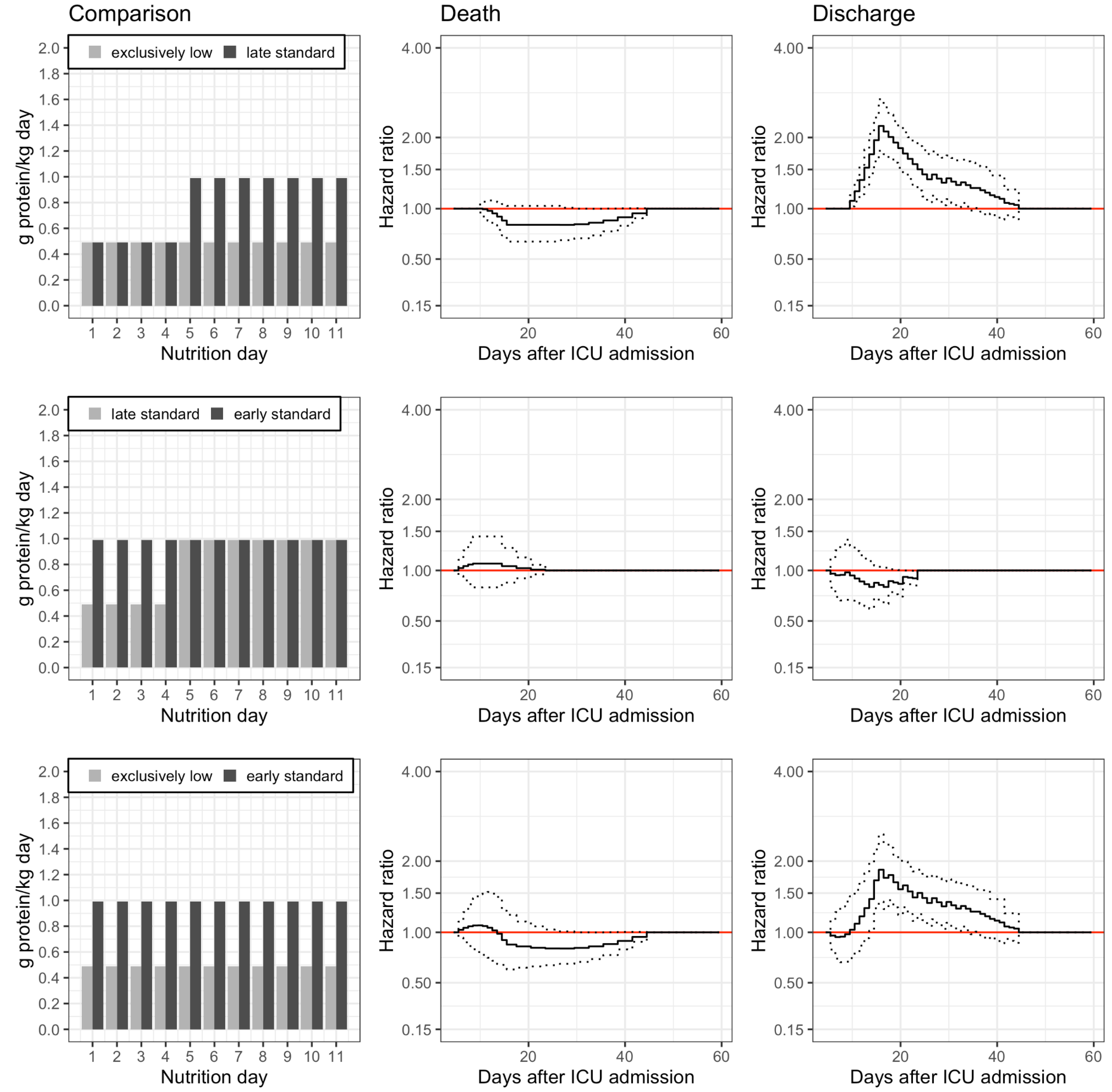


Figure S20:

Comparison of an early or late high protein intake with a standard protein intake in patients with a moderate calorie intake (30 – 70% of target) on day 3 after ICU admission

Column 1: design of diet comparisons analyzing different hypothetical protein diets (pseudo-observations) (Table 1). Protein intake reflects the median of corresponding categories (standard: 0.8 – 1.2 g protein/kg per day; high: > 1.2 g/kg per day).

Column 2 and 3: corresponding time-varying associations of different hypothetical diets with the rate of in-hospital death or live hospital discharge (cause-specific hazards).

Solid lines indicate hazard ratios (HR), hatched lines indicate corresponding 95% confidence intervals (CI) (HRs and CIs for specific time intervals after ICU admission are presented in S6 and S7 of the Additional File). Reference diet is that which provides fewer protein (e.g., an HR (and 95% CI) < 1 would indicate that rate of in-hospital death/live hospital discharge associated with the diet providing more protein was smaller). Please note that HRs (and corresponding 95% CIs) must be 1 for the first time interval between day 4 and 5 (due to the specification of the lag time), and also for time intervals, in which protein intake of both hypothetical diets is identical within the relevant time window that affects the hazard.


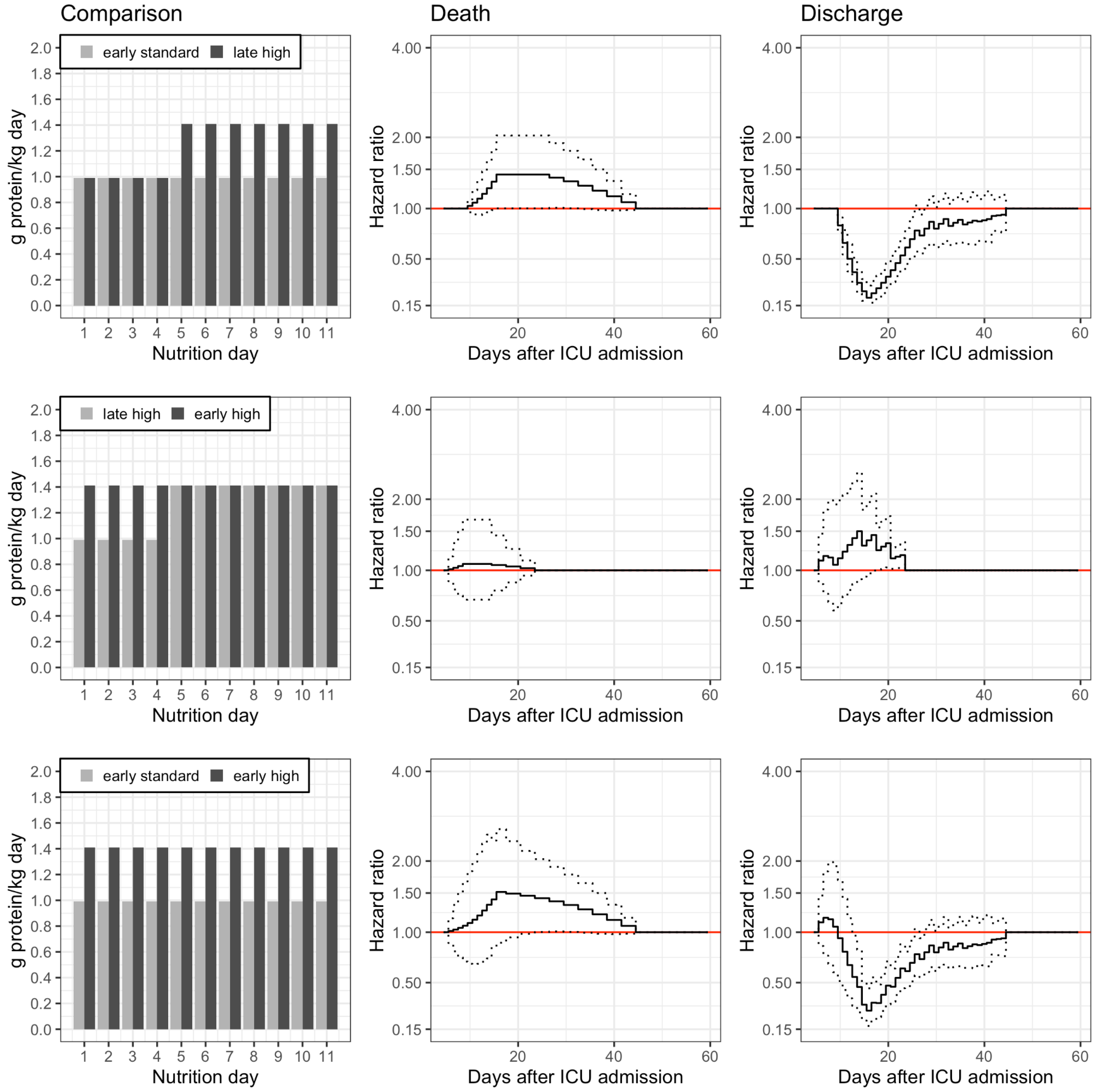


Figure S21:

Comparison of an early or late standard protein intake with a low protein intake in patients with a high calorie intake (> 70 % of target) on day 3 after ICU admission

Column 1: design of diet comparisons analyzing different hypothetical protein diets (pseudo-observations) (Table 1). Protein intake reflects the median of corresponding categories (standard: 0.8 – 1.2 g protein/kg per day; low: < 0.8 g/kg per day).

Column 2 and 3: corresponding time-varying associations of different hypothetical diets with the hazard of in-hospital death or live hospital discharge (cause-specific hazards).

Solid lines indicate hazard ratios (HR), hatched lines indicate corresponding 95% confidence intervals (CI) (HRs and CIs for specific time intervals after ICU admission are presented in Tables S4 and S6 of the Additional File). Reference diet is that which provides fewer protein (e.g., an HR (and 95% CI) < 1 would indicate that the hazard of in-hospital death/live hospital discharge associated with the diet providing more protein was smaller). Please note that HRs (and corresponding 95% CIs) must be 1 for the first time interval between day 4 and 5 (due to the specification of the lag time), and also for time intervals, in which protein intake of both hypothetical diets is identical within the relevant time window that affects the hazard


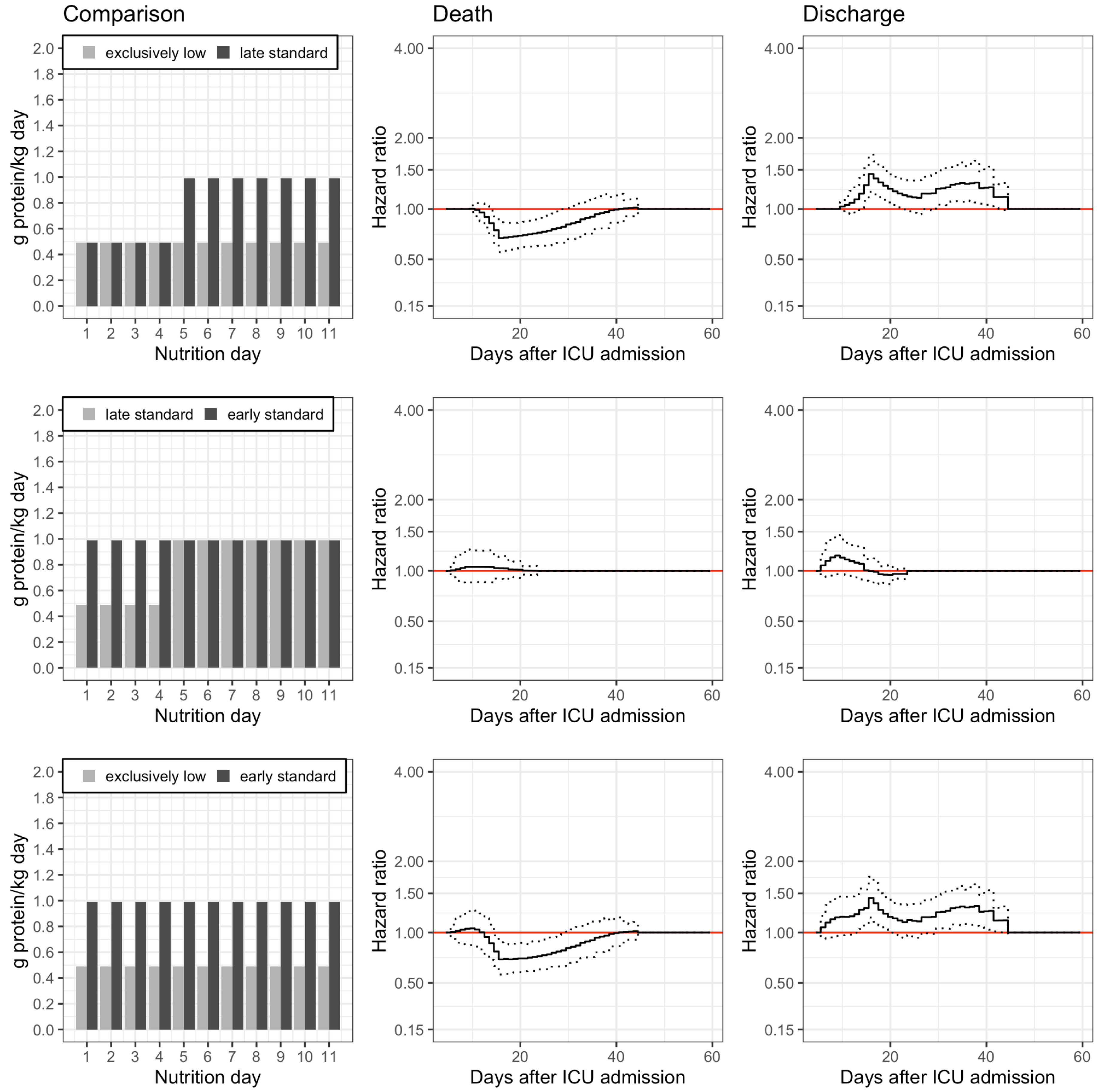


Figure S22:

Comparison of an early or late high protein intake with a standard protein intake in patients with a high calorie intake (> 70% of target) on day 3 after ICU admission

Column 1: design of diet comparisons analyzing different hypothetical protein diets (pseudo-observations) (Table 1). Protein intake reflects the median of corresponding categories (standard: 0.8 – 1.2 g protein/kg per day; high: > 1.2 g/kg per day).

Column 2 and 3: corresponding time-varying associations of different hypothetical diets with the rate of in-hospital death or live hospital discharge (cause-specific hazards).

Solid lines indicate hazard ratios (HR), hatched lines indicate corresponding 95% confidence intervals (CI) (HRs and CIs for specific time intervals after ICU admission are presented in S6 and S7 of the Additional File). Reference diet is that which provides fewer protein (e.g., an HR (and 95% CI) < 1 would indicate that rate of in-hospital death/live hospital discharge associated with the diet providing more protein was smaller). Please note that HRs (and corresponding 95% CIs) must be 1 for the first time interval between day 4 and 5 (due to the specification of the lag time), and also for time intervals, in which protein intake of both hypothetical diets is identical within the relevant time window that affects the hazard.


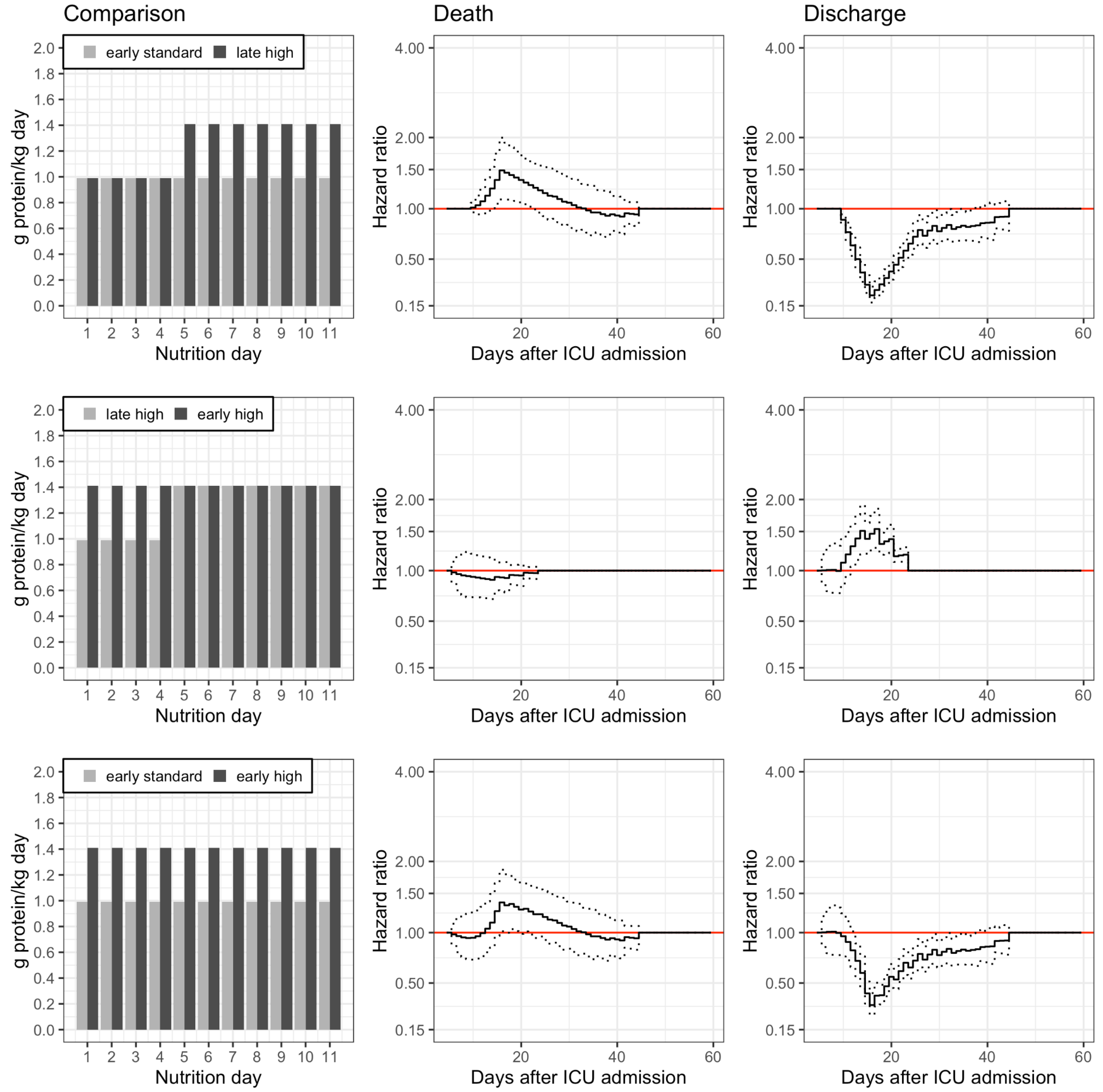


Figure S23:

Association of a variable late protein intake with outcomes in the whole cohort and in patients with various categories of calorie intake (< 30% of target, 30 – 70% of target, > 70% of target) on day 3 after ICU admission

Column 1: design of diet comparisons analyzing different hypothetical protein diets (pseudo-observations) (Table 1). Protein intake reflects the median of corresponding categories (low: < 0.8 g protein/kg per day, standard: 0.8 – 1.2 g protein/kg per day; high: > 1.2 g/kg per day).

Column 2 and 3: corresponding time-varying associations of different hypothetical diets with the rate of in-hospital death or live hospital discharge (cause-specific hazards).

Solid lines indicate hazard ratios (HR), hatched lines indicate corresponding 95% confidence intervals (CI) (HRs and CIs for specific time intervals after ICU admission are presented in S6 and S7 of the Additional File). Reference diet is that which provides fewer protein (e.g., an HR (and 95% CI) < 1 would indicate that rate of in-hospital death/live hospital discharge associated with the diet providing more protein was smaller). Please note that HRs (and corresponding 95% CIs) must be 1 for the first time interval between day 4 and 5 (due to the specification of the lag time), and also for time intervals, in which protein intake of both hypothetical diets is identical within the relevant time window that affects the hazard


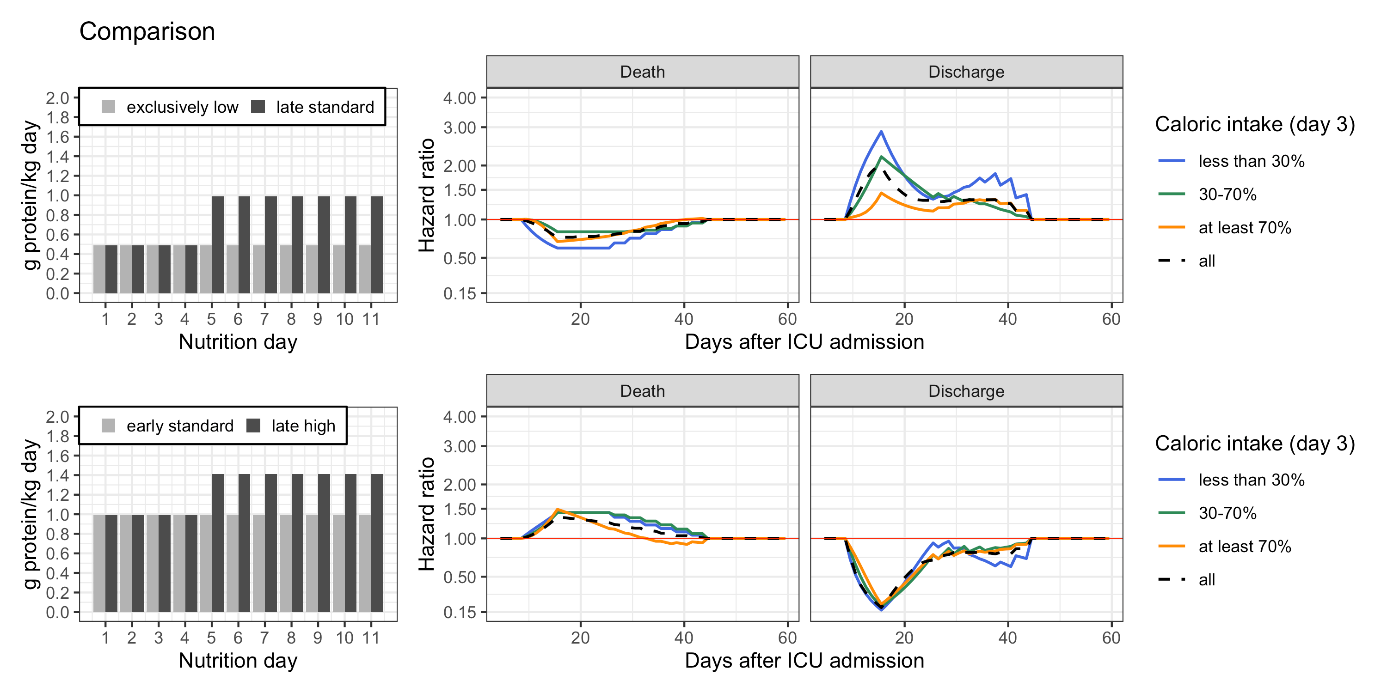

Supplement: Supplementary file 1 — Additional file 1: Supplementary information and results. [file 13054_2021_3870_MOESM1_ESM.docx]
